# Cell-free DNA release following psychosocial and physical stress in women and men

**DOI:** 10.1038/s41398-025-03242-5

**Published:** 2025-01-25

**Authors:** A. S. Limberg, F. Berg, E. Köper, C. Lindgraf, C. Gevers, R. Kumsta, E. M. Hummel, D. A. Moser

**Affiliations:** 1 Department of Genetic Psychology, Faculty of Psychology, Ruhr-University Bochum, Universitätsstraße 150, Bochum, Germany; 2 Department of Behavioural and Cognitive Sciences, Laboratory for Stress and Gene-Environment Interplay, University of Luxemburg, Porte des Sciences, Esch-sur-Alzette, Luxembourg

**Keywords:** Biomarkers, Genetics, Psychology

## Abstract

Cell-free DNA (cfDNA) is continuously shed by all cells in the body, but the regulation of this process and its physiological functions are still largely unknown. Previous research has demonstrated that both nuclear (cf-nDNA) and mitochondrial (cf-mtDNA) cfDNA levels increase in plasma in response to acute psychosocial and physical stress in males. This study further investigated these findings by testing 31 female participants (16 using oral hormonal contraception and 15 not using oral hormonal contraception), and the results were subsequently compared with those of 16 male participants. In addition, cf-nDNA and cf-mtDNA were comparatively quantified in both plasma and saliva at four time points, 2 min before and 2, 15, and 45 min after stress induction. A novel method was implemented to facilitate the straightforward collection of capillary blood by non-medical personnel for plasma analysis. While cf-mtDNA is readily detectable in body fluids due to its high copy number, the quantification of cf-nDNA is challenging due to its low abundance. To overcome this, a multiplex quantitative polymerase chain reaction (qPCR) protocol targeting *L1PA2* elements, which are prevalent in the human genome, was utilized. The analysis indicated significantly elevated levels of cf-nDNA in both plasma and saliva in all participants, irrespective of gender, following psychosocial and physical stress. Conversely, neither plasma nor saliva exhibited a consistent or stress-induced release pattern for cf-mtDNA. CfDNA is a promising biomarker that is consistently released after stress in both men and women and can be detected in both plasma and saliva. However, further research is necessary to elucidate the mechanisms of cfDNA release from specific cells and to understand its biological function in the body.

## Introduction

The study of cell-free DNA (cfDNA) has gained considerable attention in recent years, emerging as a key area of exploration in diverse clinical settings [1, 2]. CfDNA consists of DNA fragments released into the bloodstream either from the nucleus (cf-nDNA) or from the mitochondria (cf-mtDNA) [3–5] as a result of cellular processes such as necrosis, apoptosis, and NETosis [6]. Cell-free DNA is garnering increasing interest in the field of liquid biopsy, which provides a non-invasive method to monitor freely circulating genetic signatures associated with stress and disease [7]. An additional advantage of cfDNA is its presence in virtually all body fluids [3, 8–14] allowing for its detection in plasma and saliva to identify signatures related to the processing of stimuli and disease biomarkers. In this context, elevated cf-nDNA and cf-mtDNA levels are associated with trauma, sepsis, cancer, surgery, and various inflammatory diseases reviewed in [1, 2]. Moreover, cf-nDNA and cf-mtDNA are emerging as novel types of reporter molecules with growing importance in stress research [15–17]. Monitoring cf-nDNA and cf-mtDNA levels in response to psychosocial and physical stressors can thus yield valuable insights into physiological and pathological processes within the body.

Although the presence of altered levels of cf-nDNA is indicative for many acute and chronic inflammatory conditions reviewed in [18, 19], the sensitive detection of cf-nDNA as the early predictive marker is typically limited by its low concentration in body fluids such as plasma. The baseline concentration of cf-nDNA in plasma ranges approximately from 1 to 50 ng/ml (equivalent to 1–50 pg/µl) [8]. Assuming a weight of 3.23 picograms for a haploid human genome, this translates to a maximum of 15 genomic copies per microliter in healthy individuals. These low copy numbers fall below the limits of quantification (LOQ) and/or detection (LOD) for quantitative polymerase chain reaction (qPCR) when single-copy genes (SCG) [20] are employed.

At present, most studies restricted themselves mainly to cf-nDNA, while the potential of non-nuclear cell-free DNA, particularly cf-mtDNA, as novel diagnostic and prognostic biomarkers in acute and chronic conditions just recently emerged. Cf-mtDNA can either be actively released into the bloodstream directly [21], or within mitochondria [22], or packaged in vesicles [23, 24]. Since stress has been shown to increase reactive oxygen species (ROS) and lead to mitochondrial damage-associated molecular patterns (DAMPs) [25], quantifying cf-mtDNA in plasma and other body fluids offers insights into mitochondrial functionality and potential implications for various health conditions [26–28]. Some researchers hypothesize that neuroinflammatory processes lead to the release of neuronal mitochondrial DNA (mtDNA) [29], whereas others suggest immune cells as the primary source of cf-nDNA and cf-mtDNA in plasma [30, 31].

While genomic DNA exists as a diploid set per cell, the determination of mitochondrial genome copy numbers is experimentally simpler, though their origin is significantly more complex. For instance, oral mucosal or immune cells possess few dozen mitochondria, whereas high-energy-demanding cells like muscle, heart, or brain cells harbor several thousand mitochondria [32, 33]. Additionally, each mitochondrion contains multiple copies of its genome [34], with the exact number varying significantly depending on the cell type. Thus, albeit baseline levels cf-mtDNA are easier to discriminate by qPCR in comparison to cf-nDNA, it is experimentally challenging to assign a specific number of cf-mtDNA copies to a particular stimulus, as all body cells are continuously exposed to various stimuli, leading to the release of differing amounts of mtDNA. Attributing it to specific cell types or stimuli poses challenges.

In a previous study (Hummel et al. [15]) we demonstrated that establishing distinct patterns in cf-mtDNA levels in response to various stress stimuli is challenging. This contrasts with the clear patterns observed for cf-nDNA following psychosocial and physical stress [15]. In addition, the cellular origin of cf-nDNA is easier to assign based on its methylation pattern, whereas this is not yet possible for cf-mtDNA.

In the future, conducting cfDNA analyses (cf-nDNA as well as cf-mtDNA) on saliva samples, in addition to blood plasma, may prove advantageous due to their ease of collection and reduced invasiveness. However, it remains uncertain whether cf-nDNA and cf-mtDNA serve as equally reliable biomarkers in both body fluids. While initial studies have explored this direction [35], significant challenges persist in accurately quantifying cf-nDNA and identifying distinct signatures of cf-mtDNA release. Furthermore, it is essential to acknowledge that while stress can contribute to increased cf-nDNA and cf-mtDNA release, cf-nDNA and cf-mtDNA levels are influenced by contextual factors and a variety of other variables [1, 14, 36–40], including the individual’s health status, which should be carefully considered during result interpretation.

In this study, both men and women were exposed to psychosocial stress (Trier Social Stress Test; TSST) [41] and physical stress (Bicycle Ergometer Exercise Test). Given that the phase of the menstrual cycle and the use of hormonal contraceptives can influence women’s cortisol response [42, 43], reviewed in [44], and because their impact on cfDNA release is still unknown, we included two female groups (women with and without oral hormonal contraception) in the study and tested women during the luteal phase of their menstrual cycle. We utilized a novel minimally invasive method to collect capillary blood from the fingertip and concurrently collected saliva samples at four-time points to monitor cf-nDNA and cf-mtDNA levels before and after stress exposure. To quantify cf-nDNA and cf-mtDNA, we measured diluted plasma and saliva samples directly by qPCR according to a method recently described by Neuberger et al. [45]. To enhance methodological robustness, we implemented a multiplex qPCR approach and spiked the samples with DNA from a commercial lambda DNA marker to standardize and normalize PCR efficiency across all samples.

## Material and methods

The study adhered to the principles of the Declaration of Helsinki and received approval from the Ethics Committee of the Faculty of Psychology, Ruhr University Bochum (6594-BR). A total of 46 healthy participants were recruited, comprising 15 males (m) and 31 females. Among the female participants, 16 were using oral hormonal contraceptives (women with oral contraception, woc), while 15 were not using hormonal contraceptives (w; see Table 1). Only participants using contraceptives containing 0.02–0.035 mg Ethinylestradiol were included in the group woc.

**Table 1:** Age and BMI of all participants.

Group	*n*	Variable	Mean	Standard deviation
m	15	Age	21.95	2.60
		BMI	22.21	2.36
w	15	Age	21.68	2.48
		BMI	22.21	2.20
woc	16	Age	21.80	2.54
		BMI	22.22	2.36
total	46	Age	21.80	2.52
		BMI	22.38	2.31

Anthropometric data of study participants, including age (years) and body mass index (BMI, kg/m^2^), presented separately for men (m), women without oral contraception (w), and women with oral contraception (woc).

Prior to the study, all participants received a comprehensive explanation regarding objectives and potential health risks and provided written informed consent. The study comprised two experimental stress inductions: one involving psychosocial and the other physical stress. Participants were randomly assigned to undergo either of these conditions first. Using a repeated measures design, participants underwent testing a second time, two to seven days later, under the alternate stress condition. To ensure comparability with cortisol responses in men, women were tested during the luteal phase of their menstrual cycle [46]. Test sessions started at 2 p.m.

### Acute psychosocial stress

In this study, a modified version of the TSST [41] was employed to induce mild acute psychosocial stress under laboratory conditions. Participants were required to engage in a mock job interview and an arithmetic task, as validated in previous research for its efficacy in inducing a psychosocial stress response [47]. Due to COVID-19 restrictions during the testing period, transparent acrylic glass was installed in front of the interviewers. Additionally, the interviewers and the participants consistently wore face masks.

### Acute physical stress

A modified version of the physical work capacity test [48] on a bicycle ergometer was utilized as the acute physical stress test, adjusted to match the typical duration of the TSST, approximately 15 min. Following a four-minute warm-up period on the bicycle ergometer at 100 watts resistance, the workload increased by 25 watts every two minutes. Participants were instructed to maintain a cadence of 65 revolutions per minute (rpm). The exercise session concluded when participants could no longer sustain 65 rpm or reached their maximum heart rate (calculated as 220 beats per minute minus the participant’s age). Heart rate was continuously monitored by the experimenter (H3 heart rate monitor; Polar-RS800CX running watch, Kempele; Finland). On average, participants reached exhaustion after 12 min and 53 s.

### Sampling procedures

#### Blood

**Fig. 1:**
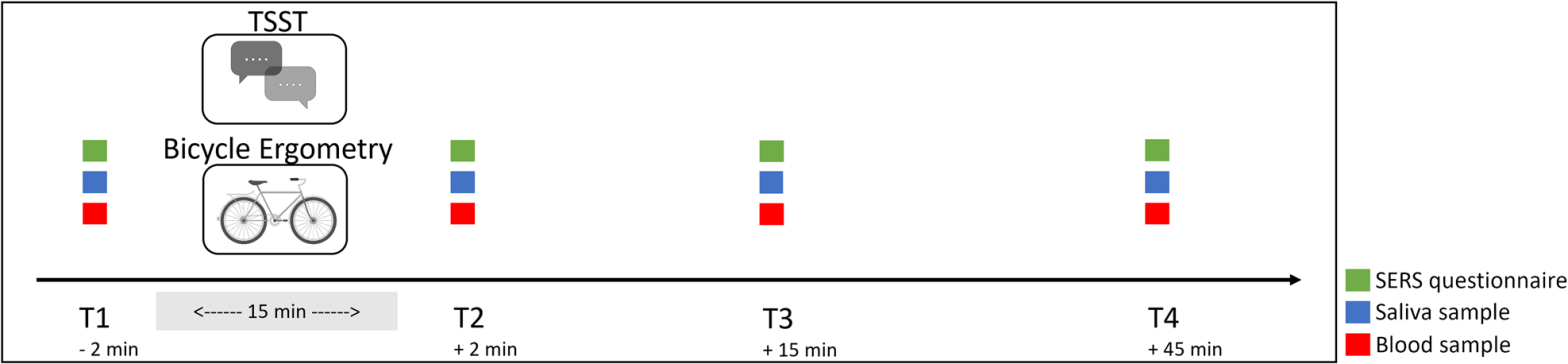
Study plan. Blood and saliva samples were collected at each time point (T1-T4). Participants were also asked to complete the Social Emotional Response Scale (SERS) questionnaire four times during each testing session. The time points were as follows: T1 = baseline (−2 min), T2 = immediately after the stressor (+2 min), T3 = anticipated peak of cf-nDNA and cf-mtDNA increase (+15 min), and T4 = expected return to baseline (+45 min). To account for the circadian rhythm of cortisol, all tests were conducted at 2 p.m.

As indicated in Fig. 1, blood and saliva samples were collected at four time points during each test session: baseline (−2 min, T1), immediately after the stressor (+2 min, T2), at the expected plateau of cf-nDNA and cf-mtDNA increase (+15 min, T3), and at the expected return to baseline (+45 min, T4). Up to 250 µl of capillary blood was drawn from the fingertip using Microvette APT 250 K2E tubes (Sarstedt, Nümbrecht; Germany), following the manufacturer´s instructions. The total blood volume was promptly transferred to a 0.5 ml tube and centrifuged at 2500 × *g* at 4 °C for 15 min. After the initial centrifugation, approximately 75% of the plasma supernatant was transferred to a new 0.2 ml tube and subjected to a second centrifugation under the same conditions as described above. Following the second centrifugation, three-quarters of the plasma were transferred to a fresh 0.2 ml tube and immediately frozen at −80 °C for subsequent cf-nDNA and cf-mtDNA quantification.

### Saliva

Saliva samples were collected from each participant at the corresponding time points prior to each blood draw (Salivette® Cortisol, Sarstedt, Nümbrecht; Germany). All subjects were instructed to keep the swabs in their mouth for 2 min without chewing. The saliva samples were processed immediately following the same protocol as described for the plasma samples. After the initial centrifugation, the remaining saliva was stored at −20 °C for subsequent analysis of salivary cortisol and salivary α-amylase.

### Questionnaire/Measurement of the social emotional response

Participants completed the Social Emotional Response Scale (SERS; Schlotz and Kumsta, 2011, unpublished) at all assessment points. The questionnaire comprises 15 items assessing tense arousal (calm, nervous, tense, intense, relaxed, satisfied), self-directed emotions (guilty, ashamed, blameful, angry with self, dissatisfied with self), and anxiety (fearful, worried). Responses were rated on a scale ranging from 1 = “not at all” to 4 = “very much”.

### Hormone measurement

To evaluate the effectiveness of the stress tests employed, salivary cortisol and salivary α-amylase activity was assessed at all four-time points. Salivary cortisol levels were analyzed using a Synergy2 plate reader (Biotek/Agilent, Santa Clara, California; USA) with a commercially available enzyme-linked immunosorbent assay (ELISA) kit for free cortisol in saliva (IBL/Tecan, Männedorf; Switzerland), following the manufacturer’s instructions. The assay demonstrated intra- and inter-assay variabilities of less than 7%. Salivary α-amylase (sAA) activity was measured using a colorimetric assay with 2-chloro-4-nitrophenyl-α-maltrotrioside (CNP-G3) as the substrate reagent, as previously described by Lorentz and colleagues [49]. This spectrophotometric assay demonstrated intra- and inter-assay variabilities of less than 4 and 5%, respectively.

### CfDNA quantification

CfDNA quantification was achieved by using qPCR and specific targets for cf-nDNA and cf-mtDNA. Quantification of cf-nDNA was conducted using multi-locus primer targeting a subfamily (*L1PA2*) of long interspersed nuclear elements *(LINE1)*, following a slightly modified protocol [45]. *L1PA2* elements, as detected by the primers and probe used here (Supplementary Table 1), occur approximately 2 374-times in the haploid human genome. This approach increases the sensitivity of the qPCR by this exact factor compared to the detection of a SCG. Cf-mtDNA was quantified using custom-designed mtDNA primers as detailed in Supplementary Table 1.

To control for potential inhibitors and to normalize the samples, 250 copies of a commercially available Lambda-BstEII marker (λ DNA-BstEII; New England BioLabs, Ipswich, Massachusetts; USA) were added per microliter of PCR cocktail, corresponding to 5000 copies per 20 µl reaction. The use of this commercially available DNA size standard facilitates straightforward and standardized quality control, potentially contributing to the standardization of cf-nDNA and cf-mtDNA analysis in the future.

Multiplex qPCR was performed in a final volume of 20 µl using SsoAdvanced Universal Probe Supermix (Biorad, Hercules, California; USA), primers, and probes as detailed in Supplementary Table 1. Each reaction included 1.5 µl of 1:10 water-diluted plasma or saliva samples. Absolute quantification was achieved by comparing unknown samples to a serial dilution of known amounts of target DNAs ranging from 2.5 × 10^8^ to 2.5 × 10^2^ copies/µl for all three targets analysed.

The copy number of the spike-in control was determined based on the initial concentration (500 ng/ml) of the commercially available λ-DNA-BstEII length standard (NEB#B7025) and the length of the lambda genome of 48 502 bp using the DNA Copy Number and Dilution Calculator (https://www.thermofisher.com). A 229 bp Lambda DNA BstEII restriction fragment was detected using the primers shown in Supplementary Table 1. Two other DNA standards, a custom-made 114 bp *L1PA2* family standard (Supplementary Table 1) and a custom-made 84 bp mitochondrial DNA standard (Supplementary Table 1), were purchased at a concentration of 100 µM from MWG Eurofins (Ebersberg; Germany). The copy number for each fragment was calculated based on its concentration and length. Subsequently, a pool of all standards was prepared with an initial concentration of 2.5 × 10^10^ copies/µl for each fragment and serially diluted 100-fold (from 2.5 × 10^8^ to 2.5 × 10^2^ copies/µl; for standard curves see Supplementary Fig. 8). QPCR was conducted on a CFX 384 real-time cycler (Biorad, Hercules, California; USA) with an initial heat activation of 95 °C for 5 min, followed by 39 cycles of 95 °C for 15 s, 55 °C for one minute, and a plate read.

### Inter-run calibration and normalization

The lambda spike-in was utilized to normalize the data by determining the average lambda copy number per plate. Subsequently, a correction factor was computed by dividing the average lambda copy number by the sample-specific lambda copies.

Following previously described protocols [45, 50] a custom inter-run calibration protocol was established using serial dilutions of standards on each plate as inter-run calibrators for absolute quantification. As a part of this procedure, the mean deviation of standards on one plate from the mean deviation across all plates is calculated as a percentage. This value serves to derive a correction factor for inter-run variability specific to each plate.

### Statistical analysis

Statistical analysis of the samples was conducted using R (version 4.3.1) and RStudio (version 2023.12.1 + 402). Prior to data collection, a power analysis was performed using G*Power 3 [51, 52] to determine an adequate sample size capable of detecting potential effects of acute stress on cf-nDNA and cf-mtDNA concentration. The effect size was set at 0.89, and the correction factor for repeated measures was set to 0.3, based on previous findings by Hummel et al. [15]. Additionally, the power was set to 0.95 with a level of significance α = 0.05.

To assess differences over time, between conditions, and among groups, a three-way repeated measures analysis of variance (rmANOVA) was employed. Violations of the sphericity assumption were addressed through adjustment of the degrees of freedom. Normality was assessed using Shapiro-Wilk’s test and visually inspected through Q-Q plots. Specific post-hoc comparisons between baseline and post-stressor levels were conducted by pairwise comparisons. Due to deviations from normality, levels of cf-nDNA, cf-mtDNA, salivary cortisol, and salivary α-amylase were logarithmically transformed. Spearman correlations were calculated and adjusted using Bonferroni’s correction to explore relationships between changes in cf-nDNA, cf-mtDNA, salivary cortisol, salivary α-amylase, and peak heart rate. Some participants were excluded from certain analyses due to data quantification issues.

Based on this statistical approach, the power analysis indicated that a sample size of 12 participants is required for the rmANOVA.

## Results

### Changes in the salivary cortisol concentrations

Data from 45 out of 46 participants was included in the analysis of salivary cortisol concentrations (15 m, 14 w, and 16 woc). Supplementary Fig. 1 shows the measured salivary cortisol concentration in nmol/l before and after psychosocial and physical stress induction for all participants (Supplementary Fig. 1A) and for the three groups (m, w, and woc/ Supplementary Fig. 1B). Salivary cortisol levels increased by 1.6-fold following exposure to the psychosocial stressor (T2) and by 1.9-fold following exposure to the physical stressor (T2) (refer to Supplementary Fig. 1). The rmANOVA revealed a significant main effect of the time of measurement (*F*(1.68, 70.64) = 23.75, *p* < 0.001, η2_p_ = 0.36) and a significant interaction between the time of measurement and the type of stressor (*F*(1.69, 71.04) = 5.19, *p* = 0.01, η^2^_p_ = 0.11). No other main effects reached statistical significance.

Post-hoc tests examining the interaction effect within stressor types revealed significant general differences between time points for both psychosocial stress (*F*(1.56, 68.5) = 16.6, *p* < 0.001, η^2^_p_ = 0.27) and physical stress (*F*(1.69, 74.5) = 16.2, *p* < 0.001, η^2^_p_ = 0.27).

### Changes in salivary α-amylase levels

For the analysis of salivary α-amylase levels, data from all 46 participants was included (15 m, 15 w, and 16 woc). The measured salivary α-amylase activity [U/ml] is presented in Supplementary Fig. 2. The figure shows the salivary α-amylase activity before and after psychosocial and physical stress introduction for all participants (Supplementary Fig. 2A) and divided by group (m, w and woc/ Supplementary Fig. 2B). For the psychosocial stressor, salivary α-amylase levels decreased by a factor of 1.2 from T1 to T3. Conversely, following the physical stressor (T2), salivary α-amylase levels increased by 1.2-fold (see Supplementary Fig. 2A). Statistical analysis revealed a significant main effect of time point (*F*(3, 129) = 16.27, *p* < 0.001, η^2^_p_ = 0.28) and a significant interaction effect of stressor type by time point (*F*(3, 129) = 6.21, *p* < 0.001, η^2^_p_ = 0.13).

Post-hoc analyses further indicated non-specific significant differences between time points for both the psychosocial stress condition (*F*(2, 112) = 3.07, *p* = 0.04, η^2^_p_ = 0.06) and the physical stress condition (*F*(3, 135) = 16.6, *p* < 0.001, η^2^_p_ = 0.27).

### Changes in plasma cfDNA concentrations

#### Plasma cf-nDNA

**Fig. 2:**
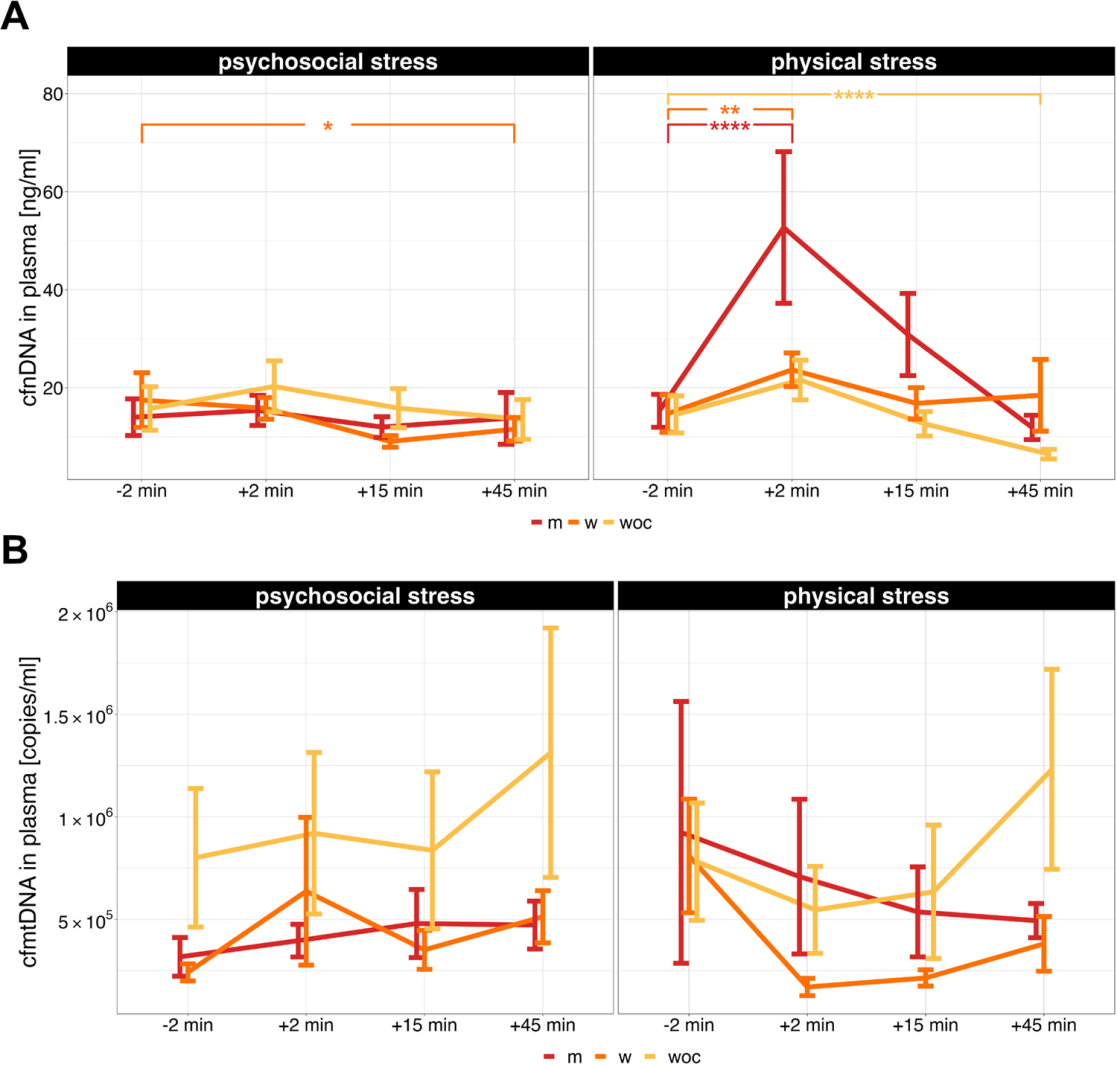
Levels of cf-nDNA and cf-mtDNA in plasma. **A** Plasma cf-nDNA concentration before and after the onset of acute psychosocial and physical stress for the three groups (m = men, w = women, woc = women on hormonal contraception). **B** cf-mtDNA concentration in plasma before and after the induction of acute psychosocial and physical stress for the three groups (m, w, woc). Plasma cf-nDNA is presented in ng/ml over time, while cf-mtDNA is presented in copies/ml. Since the weight of a haploid human genome is estimated to be approximately 3.23 pg, a concentration of 1 ng cf-nDNA/ml corresponds to roughly 310 genomic copies/ml. Error bars represent the standard error of the mean. Plasma cf-nDNA levels generally showed significant changes over time, as well as significant differences between psychosocial and physical stress conditions. Additionally, rmANOVAs revealed significant interaction effects between stress types and time points, as well as between stress types and groups. A significant three-way interaction was also observed between groups, stress types, and time points. Follow-up analysis showed significant differences in plasma cf-nDNA levels between the psychosocial and physical stress conditions. Furthermore, a significant main effect was found for changes over time in male, female, and female participants using oral hormonal contraceptives. The rmANOVA for plasma cf-mtDNA revealed a main effect for changes over time and an interaction between stress types and time points. Post-hoc tests indicated significant differences in plasma cf-mtDNA levels between psychosocial and physical stress conditions. *p < 0.05, **p < 0.01, ***p < 0.001, ****p < 0.0001.

The analysis of cf-nDNA included data from 44 of the 46 participants (15 m, 13 w, 16 woc). Figure 2A shows the cf-nDNA concentration [ng/ml] in plasma before and after psychosocial and physical stress onset divided by group (m, w and woc) while Supplementary Fig. 3A presents plasma cf-nDNA concentration for all participants. For the psychosocial stress paradigm, the descriptive analysis showed low increase of cf-nDNA levels. Cf-nDNA concentration for the physical stressor peaked at T2 and increased by a factor of 2.2 followed by a steady decrease (Supplementary Fig. 3A and Supplementary Tables 2 and 3).

However, cf-nDNA levels changed significantly over the course of the study (main effect of time: *F*(2.47, 101.39) = 27.44, *p* < 0.001, η^2^_p_ = 0.40). There were significant differences in cf-nDNA levels (both in magnitude and response dynamics) between the two stressors (main effect of stressor type: *F*(1, 41) = 5.59, *p* = 0.02, η^2^_p_ = 0.12) and over time between the two stressors (time × stressor type interaction: *F*(3, 123) = 7.86, *p* < 0.001, η^2^_p_ = 0.16; Supplementary Fig. 3A).

Post hoc analyses indicated that these differences were evident for both the psychosocial and physical stressors (psychosocial stressor *F*(3, 129) = 6.00, *p* < 0.001, η^2^_p_ = 0.12; physical stressor *F*(2, 57.11) = 28.20, *p* < 0.001, η^2^_p_ = 0.40). Pairwise comparisons showed a significant increase of cf-nDNA after the psychosocial stressor (psychosocial stressor T1 to T2 *t*(43) = −2.11, *p* = 0.041) and the physical stressor (physical stressor T1 to T2 *t*(43) = −5.77, *p* < 0.001). Additionally, the physical stressor showed a significant change in cf-nDNA from T1 to T4 (*t*(43) = 3,28, *p* = 0.002; see Supplementary Fig. 3A).

Furthermore, there were significant differences in cf-nDNA levels between groups across time for both stress conditions (group × stressor: *F*(2, 41) = 3.49, *p* = 0.04, η^2^_p_ = 0.15; time × group × stressor interaction: *F*(6, 123) = 2.31, *p* = 0.04, η^2^_p_ = 0.10). The main effect of the group and the interaction between group and time were not significant. Group-specific rmANOVAs were conducted for further exploration. All three groups exhibited significant changes in cf-nDNA over time (main effect of time: males *F*(3, 42) = 13.20, *p* < 0.001, η^2^_p_ = 0.49; females *F*(1.55, 18.60) = 4.89, *p* = 0.03, η^2^_p_ = 0.29; females with oral hormonal contraception *F*(3, 45) = 12.80, *p* < 0.001, η^2^_p_ = 0.46; see Fig. 2A).

Additionally, the cf-nDNA levels differed significantly between male participants across the two stressors (main effect of stressor: males *F*(1, 14) = 10.20, *p* = 0.006, η^2^_p_ = 0.42). The concentration of cf-nDNA to both stressors also varied over time within the male group and among females using oral hormonal contraception (interaction of time × stressor: males *F*(3, 42) = 7.18, *p* < 0.001, η^2^_p_ = 0.34; females with oral contraception *F*(3, 45) = 3.32, *p* = 0.03, η^2^_p_ = 0.18; see Fig. 2A). Pairwise comparisons revealed a significant change in cf-nDNA levels among the female participants (w) during the psychosocial stress condition from T1 to T4 (*t*(12) = 2.37, *p* = 0.036). For the physical stressor, both male and female participants exhibited significant changes in cf-nDNA after the stressor (male participants, physical stressor from T1 to T2, *t*(14) = −4.99, *p* < 0.001; female participants without hormonal contraception, physical stressor from T1 to T2, *t*(12) = −3.27, *p* = 0.007). For female participants using hormonal contraception cf-nDNA changed significantly from T1 to T4 of the testing session (*t*(15) = 4.15, *p* < 0.001; see Fig. 2A).

#### Plasma cf-mtDNA

The analysis of plasma cf-mtDNA included data from 42 of the 46 participants (14 m, 13 w, 15 woc). The concentration of cf-mtDNA in plasma [copies/ml] before and after psychosocial and physical stress onset is illustrated in Fig. 2B split by group (m, w and woc) and in Supplementary Fig. 3B for all participants. Descriptive analysis of plasma cf-mtDNA showed an increase over time for the psychosocial stressor with a peak at T4. Throughout the test session, within the psychosocial stress paradigm, cf-mtDNA levels increased by a factor of 1.6. The data showed a peak in cf-mtDNA at T1 for the physical stressor. Cf-mtDNA decreased by a factor of 1.7 from T1 to T2 following physical stress (Supplementary Fig. 3B, Supplementary Tables 4 and 5). This initial decrease (T2 to T3) was subsequently followed by an increase in cf-mtDNA levels at T4 (Supplementary Fig. 3B). The rmANOVA revealed a significant main effect of time (*F*(3, 117) = 4.4, *p* = 0.006, η^2^_p_ = 0.1) and an interaction between time and stressor (*F*(3, 117) = 4.74, *p* = 0.004, η^2^_p_ = 0.12). Subsequent post-hoc tests revealed unspecific significant differences in plasma cf-mtDNA after psychosocial stress (*F*(3, 123) = 3.58, *p* < 0.016, η^2^_p_ = 0.08) and after physical stress (*F*(3, 123) = 5.51, *p* < 0.001, η^2^_p_ = 0.12). Further analysis using pairwise comparisons revealed a significant increase of cf-mtDNA during the psychosocial stress condition after the stressor (psychosocial stressor from T1 to T2, *t*(41) = −3.03, *p* = 0.004). Besides that cf-mtDNA also increased significantly over the course of the testing session with the psychosocial stressor (psychosocial stressor from T1 to T4, *t*(41) = −2.81, *p* = 0.008). Moreover, cf-mtDNA also showed a significant decrease after the physical stressor (physical stressor T1 to T2, *t*(41) = 2.54, *p* = 0.015; see Supplementary Fig. 3B). There were no significant differences between groups for cf-mtDNA in plasma (Fig. 2B).

### Changes in salivary cfDNA concentrations

#### Salivary cf-nDNA

**Fig. 3:**
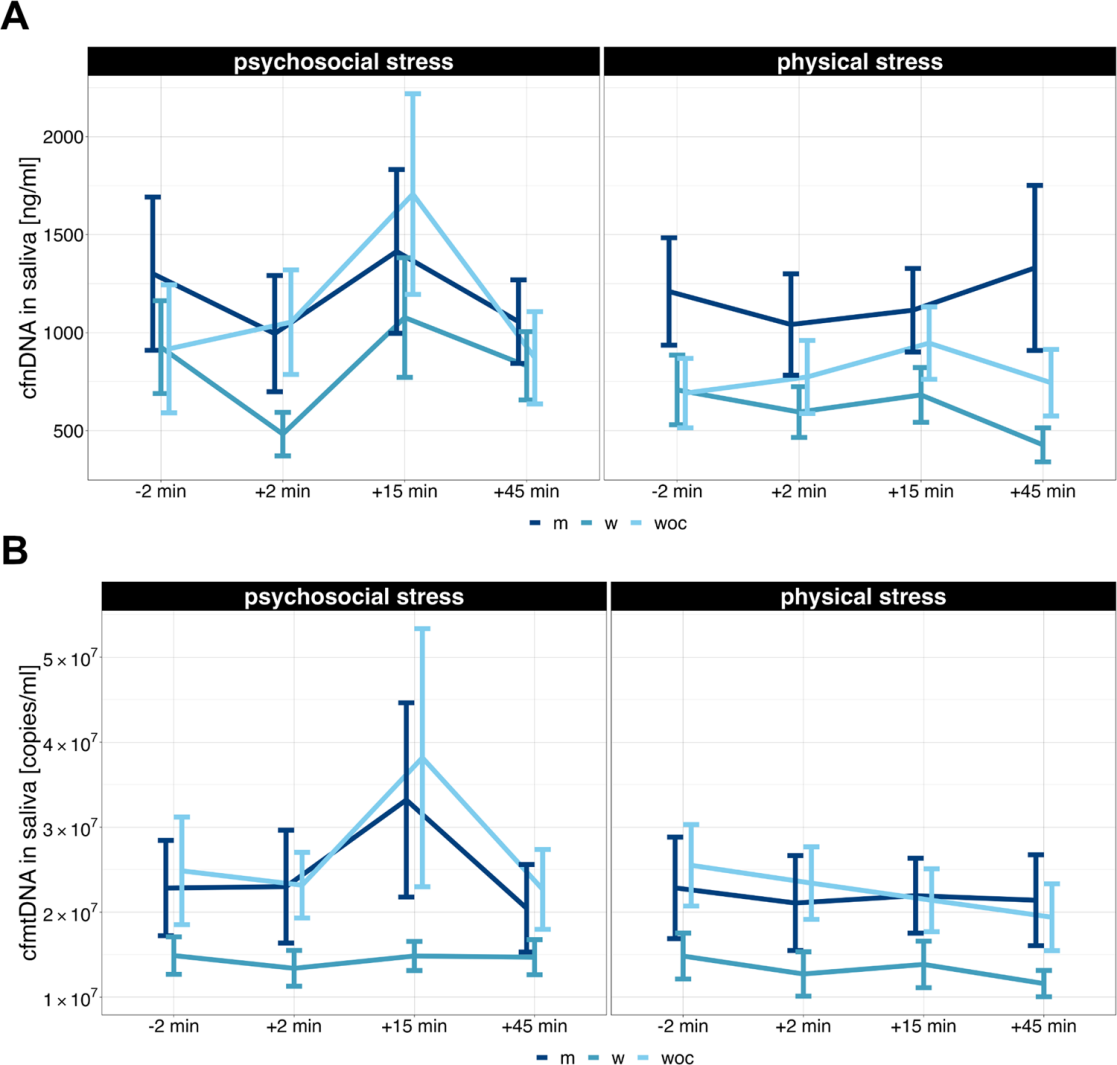
Levels of cf-nDNA and cf-mtDNA in saliva. **A** Salivary cf-nDNA concentrations before and after the induction of acute psychosocial and physical stress for the three groups (m = men, w = women, woc = women on hormonal contraception). **B** Salivary cf-mtDNA concentrations before and after the onset of acute psychosocial and physical stress for the three groups (m, w, woc). Salivary cf-nDNA is presented in ng/ml over time, while cf-mtDNA is presented in copies/ml. Since the weight of a haploid human genome is estimated to be approximately 3.23 pg, a concentration of 1 ng cf-nDNA/ml corresponds to roughly 310 genomic copies/ml. Error bars represent the standard error of the mean. rmANOVAs for salivary cf-nDNA and cf-mtDNA revealed a main effect for changes over time in cf-nDNA concentration, but no significant effects for cf-mtDNA.

Salivary cf-nDNA analysis included data from 42 of the 46 participants (13 males, 15 females, 14 using oral contraceptives). The concentration of salivary cf-nDNA [ng/ml] before and after psychosocial and physical stress induction is shown in Fig. 3A split by group (m, w, and woc) and in Supplementary Fig. 4A for all participants. Descriptive analysis revealed a peak in salivary cf-nDNA concentration at T3 for the psychosocial stressor, whereas during the physical stress condition, the salivary cf-nDNA concentration remained relatively stable (Supplementary Fig. 4A, and Supplementary Tables 6 and 7). Specifically, salivary cf-nDNA increased by a factor of 1.3 for the psychosocial stressor (Supplementary Fig. 4A). RmANOVA indicated a significant main effect only for the time of measurement (*F*(2, 89.36) = 4.28, *p* = 0.01, η^2^_p_ = 0.10). Pairwise comparisons demonstrated a significant increase in cf-nDNA from T1 to T3 (*t*(83) = −2.69, *p* = 0.009; see Supplementary Fig. 5). No significant group-specific effects were observed (Fig. 3A).

#### Salivary cf-mtDNA

Salivary cf-mtDNA analysis encompassed data from 43 of the 46 participants (13 males, 15 females, 15 using oral contraceptives). Figure 3B displays the salivary cf-mtDNA levels [copies/ml] before and after psychosocial and physical stress onset for each of the three groups (m, w, and woc), while Supplementary Fig. 4B shows the salivary cf-mtDNA levels for all participants. Descriptive analysis revealed a 1.5-fold increase in salivary cf-mtDNA levels at T3 following psychosocial stress, with comparable levels observed after physical stress (Supplementary Fig. 4B, Supplementary Tables 8 and 9). However, no statistically significant effects were found (Fig. 3B).

#### Correlations between cf-nDNA, cf-mtDNA, stress hormone levels, and heart rate

In addition to the rmANOVAs, pre-post differences in all cfDNA markers (cf-nDNA and cf-mtDNA in plasma and saliva) were computed and correlated with pre-post differences in salivary cortisol and salivary α-amylase levels. Furthermore, maximum heart rate was correlated with increases in cfDNA markers during the exercise condition. The analysis encompassed data from 36 participants (12 males, 11 females, 13 using oral contraceptives). However, none of the correlations achieved statistical significance.

## Discussion

The aim of this study was to investigate the release of cf-nDNA and cf-mtDNA in response to acute psychosocial and physical stress conditions in men and women, assessed simultaneously in both plasma and saliva. Plasma samples were generated using a novel, minimally invasive capillary blood draw method suitable for broad application, and cf-nDNA and cf-mtDNA were quantified directly by qPCR without further purification or extraction.

### cf-nDNA in plasma

Across all participant groups, a consistent pattern of plasma cf-nDNA release before and after stress exposure was observed, indicating a uniform response to upstream signals across genders (Fig. 2A).

Overall, the TSST elicited a modest increase in cf-nDNA levels in both men and women (1.1-fold increase), whereas physical exercise resulted in a more pronounced 2.2-fold increase (Supplementary Fig. 3A). Specifically, during the psychosocial stress task, men exhibited a minor change in cf-nDNA concentration (1.1-fold increase). For female participants in the TSST, those using oral hormonal contraception showed a slight increase (1.3-fold), while those not using hormonal contraception showed a slight decrease (0.9-fold decrease; see Fig. 2A).

The lower cf-nDNA increase observed under psychosocial stress compared to previous studies [15–17] may be attributed to differences in study design. Firstly, the participants in our study were predominantly university students, including psychology majors, who likely had some familiarity with the TSST concept, potentially blunting anticipatory stress effects. This interpretation is supported by the modest changes in subjective stress levels and stress-related emotions reported (Supplementary material: SERS results; Supplementary Figs. 6 and 7). Moreover, the TSST was conducted with participants wearing face masks, mandated by COVID-19 regulations, which may have attenuated the perceived stress intensity.

Physical stress resulted in a greater overall increase of cf-nDNA compared to psychosocial stress. However, the increased levels of cf-nDNA under physical stress conditions were primarily driven by changes observed in men, who exhibited a 3.4-fold increase in cf-nDNA. Men sustained significantly longer periods on the bicycle ergometer, pedaled at higher resistance levels, and achieved notably elevated salivary cortisol levels compared to female participants. Women not using hormonal contraceptives showed a 1.6-fold increase in cf-nDNA after exercise, while women using oral hormonal contraceptives exhibited a 1.5-fold increase (see Fig. 2A). Despite reaching heart rates like those of men, women using hormonal contraceptives engaged in significantly shorter and less intensive exercise bouts. Additionally, the physical exercise was conducted using a bicycle ergometer, which typically induces less physical stress and lower energy expenditure compared to treadmill tests [53, 54]. It is also plausible that psychology students, on average, have lower physical fitness levels compared to sports students recruited in other studies [15, 16].

For the first time, women were directly compared to men under two distinct stress conditions, revealing that women reached their physical limits significantly faster and exhibited lower levels of salivary cortisol and cf-nDNA (Supplementary Figs. 1A and 2A). Our findings align with previous studies reporting higher cfDNA levels in men compared to women [55–58]. Although the menstrual cycle did not seem to influence cfDNA levels at rest in females [59–61], evidence suggests that the balance of female sex hormones may modulate cfDNA release after moderate exercise [61]. In our study, all women in group “w” were tested during the luteal phase, characterized by elevated levels of female sex hormones [62], potentially influencing cfDNA release in this cohort.

### cf-mtDNA in plasma

The analysis of cf-mtDNA in plasma revealed significant differences in cf-mtDNA levels following the psychosocial stressor as well as the physical stressor. Interestingly, women using oral hormonal contraception exhibited the highest cf-mtDNA levels following both stressors, despite considerable variability in the data (Fig. 2B, Supplementary Tables 4 and 5). Further research is required to identify the specific cell types responsible for releasing cf-mtDNA in response to stress, to understand the physiological triggers underlying this release, and to clarify the biological functions of circulating cf-mtDNA.

### cfDNA in saliva

In addition to analyzing cf-nDNA and cf-mtDNA in plasma, we also assessed these markers in saliva. Generally, the levels of cf-nDNA and cf-mtDNA were significantly higher in saliva compared to plasma (Figs. 2 and 3; Supplementary Figs. 3 and 4). In contrast to cf-nDNA in plasma, cf-nDNA in saliva initially decreased after both stressors but subsequently increased by the end of the experiment (Supplementary Fig. 4A). It remains uncertain whether these effects are consistently related to stress. Cf-mtDNA concentration in saliva increased 15 min after psychosocial stress, driven mainly by outliers, whereas it tended to decrease slightly after physical stress (Supplementary Fig. 4B). Although previous studies, including ours, have linked mtDNA in monocytes and cf-mtDNA in circulation to inflammatory processes, mental disorders, and stress [15, 63–65], direct measurements of cf-mtDNA in plasma and saliva did not confirm such associations with stress. The inconsistent and variable cf-mtDNA values, seemingly independent of stress stimuli, are also reported by others [15, 28, 35, 65–68]. Overall, data on cf-mtDNA levels in plasma are highly heterogeneous in the literature, and there is limited information on cf-mtDNA in saliva. Conflicting findings across studies may stem from differences in purification and quantification methods [28, 65–68], as well as significant intra- and inter-individual variability in cf-mtDNA levels among study participants and controls.

Unlike cf-nDNA, there may not be a universal cf-mtDNA signature linked to a specific stimulus, given the variation in mitochondrial content and mtDNA copy numbers among human cells. The release of cf-mtDNA can also be influenced by different stimuli [32–34]. Some researchers propose that cf-mtDNA serves as a link between the brain and immune system in neuro-immunological diseases [29], while others suggest it originates mainly from immune cells. Recent evidence indicates that elevated cfDNA after exercise predominantly originates from mature polymorphonuclear neutrophils, with a minor contribution from cardiomyocytes [69]. Differences in quantification methods may account for variations in cf-mtDNA levels observed across studies, particularly in plasma. Michelson et al. [70] recently introduced a method for direct cf-nDNA and cf-mtDNA measurement in serum, plasma, and saliva. The protocol uses a detergent-containing buffer to enhance mtDNA quantification by lysing circulating mitochondria. However, their use of a single-copy gene (*beta-2-microglobulin; B2M*) as a standard for cf-nDNA quantification resulted in values below the limit of quantification (LOQ) due to its low concentration in body fluids and additional dilution by the lysis buffer [70]. Consequently, no direct comparisons could be made regarding cf-nDNA levels between plasma and saliva. Nevertheless, Michelson et al. [70] reported higher levels of cf-mtDNA in plasma compared to our findings, supporting the effectiveness of their protocol in lysing circulating mitochondria. It should be noted that residual cellular material in plasma, such as anucleate platelets, could be lysed and quantified using this protocol, which may lead to an overestimation of cf-mtDNA levels.

In the future, it would be beneficial to further develop and simplify the methodology for cfDNA analysis by directly measuring cfDNA in plasma or saliva samples, for example, using fluorometric methods. However, the low concentrations of cfDNA and the background fluorescence from plasma proteins must be considered. Additionally, employing a fluorometric method would not enable differentiation between cf-nDNA and cf-mtDNA.

Saliva measurements consistently showed mean cf-nDNA levels under baseline conditions (T1) to be 60 times higher than in plasma (for data see: Supplementary Tables 2 and 3 for plasma cf-nDNA, and Supplementary Tables 6 and 7 for cf-nDNA in saliva). Notably, baseline cf-mtDNA levels in saliva were found to be approximately 40 times higher than in plasma and exhibited considerable variability even under baseline conditions, ranging from 5 × 10^5^ copies/ml to 9 × 10^7^ copies/ml across all subjects studied (for data see: T1 in Supplementary Tables 8 and 9), without a clear stress-related trend. This variability is unexpected given the low mitochondrial content and mtDNA copy numbers in mucosal cells. This suggests that mtDNA in saliva may enter through plasma filtrate or be actively secreted by salivary glands and accumulate in the oral cavity. Further investigation into the methylation signatures of cf-nDNA and cf-mtDNA in plasma and saliva, as recently explored [15, 71–74], could provide valuable insights into their origins and functions.

Based on our findings, the question remains unresolved regarding whether increased cf-nDNA and cf-mtDNA levels stem from enhanced release, diminished degradation, reduced elimination from circulation, or a combination thereof. One potential factor influencing this dynamic is the differential presence of DNase in the analyzed body fluids. Plasma, for instance, contains more than twice the amount of DNase compared to saliva [75], potentially contributing to elevated cfDNA levels and an extended half-life of approximately 2 h for cfDNA in saliva [76].

It is noteworthy that cf-nDNA and cf-mtDNA levels increase not only under pathological conditions but also following physical exertion [15, 16]. This suggests that cf-nDNA and cf-mtDNA may have roles in intercellular signaling, in addition to its functions related to apoptosis and clearance. Presumably, the release of DNA molecules, which entails an energy expenditure equivalent to one molecule of ATP per nucleotide, serves a purposeful function rather than constituting a wasteful expenditure of energetically expensive DNA units. Future investigations should explore whether cf-nDNA and cf-mtDNA might serve as ancient communication molecules akin to bacterial plasmids involved in conjugation. However, given its extensive fragmentation, the capacity of cfDNA to convey transcriptionally active units is dubious; yet it remains conceivable that cfDNA could induce epigenetic modifications in recipient cell genomes through recombination events.

Both psychosocial stress and physical exertion impose substantial demands on the organism, necessitating various adaptive mechanisms across different biological levels. Epigenetic alterations in DNA methylation patterns and chromatin modifications specific to cell types occur [77, 78] within the cell nucleus complemented by miRNA-mediated regulation of protein expression in the cytosol [6, 79–81]. Furthermore, single nucleotide polymorphisms (SNPs) and environmental factors exert significant influence on the epigenome and health outcomes [65, 82–84], alongside the impact of stressful life events and diseases on RNA and protein expression levels [85–88]. Nonetheless, the issue of cellular tissue and body fluids selection in the context of psychobiological research remains contentious and warrants further exploration [64, 89–92].

In conclusion, it is crucial to recognize that cf-nDNA and cf-mtDNA represent just one facet of the intricate cellular and extracellular systems involved in maintaining homeostasis. The study of cf-nDNA and cf-mtDNA in the context of stress responses promises deeper insights into the biological mechanisms underlying the complex multisystemic stress response. Our study underscores that plasma cf-nDNA serves as a robust indicator of stress response following acute psychosocial and physical stress in both men and women. Nevertheless, we did not observe a stress-induced signal for plasma cf-mtDNA or for cf-nDNA and cf-mtDNA in saliva. Continued research efforts are essential to fully elucidate the potential of cf-nDNA and cf-mtDNA as biomarkers for stress and to expand our understanding of their origins and characteristics.

## Supplementary information

Supplementary material: Limberg et al. (2025)_ Cell-free DNA release following psychosocial and physical stress in women and men

## Data Availability

The cf-nDNA and cf-mtDNA datasets generated during the current study are included in the Supplementary Material. Hormone datasets are available from the corresponding author upon request.

## Code availability

The R code used for the statistical analysis of the data is available from the corresponding author upon reasonable request.
