# Supplementary material for: Cell-free DNA release following psychosocial and physical stress in women and men
Source: Transl Psychiatry. 2025 Jan 25;15:26. doi: 10.1038/s41398-025-03242-5 (PMC11763022; doi:10.1038/s41398-025-03242-5)
Supplement: Supplementary file 1 — Supplementary material: Limberg et al. (2025)_ Cell-free DNA release following psychosocial and physical stress in women and men [file 41398_2025_3242_MOESM1_ESM.docx]

Supplementary material:

Cell-free DNA release following psychosocial and physical stress in women and men

A.S. Limberg^§^, F. Berg^§^, E. Köper, C. Lindgraf, C. Gevers, R. Kumsta, E.M. Hummel & D.A. Moser^*^

-----------------------------------------------------------------------------------------------------------------

**Table of Contents** **1**

**Supplementary Figures** **2**

Supplementary Figure 1: Salivary Cortisol Concentration 2

Supplementary Figure 1: Salivary α-amylase Activity Levels 3

Supplementary Figure 2: Plasma Levels of cf-nDNA and cf-mtDNA 4

Supplementary Figure 4: Quantification of cf-nDNA and cf-mtDNA in Saliva 5

Supplementary Figure 5: Quantification of cf-nDNA in Saliva 6

**SERS Results** **7**

Changes in Subjective Stress Perception 7

Tense Arousal 7

Anxiety and Self-Directed Emotions 7

Supplementary Figure 6: Summary of SERS Questionnaire Responses 8

Supplementary Figure 7: Distribution of Tense Arousal Responses in SERS 9

**Standard Curves 10**

Supplementary Figure 8: Standard curves for L1PA2 (cf-nDNA), mtDNA (cf-mtDNA), 10

and Lambda-DNA BstEII 229 bp fragment (spike-in)

**Supplementary Tables** **11**

Supplementary Table 1: Primers, probes, and standards 11

Supplementary Table 2: Plasma cf-nDNA before and after psychosocial stress induction 12

Supplementary Table 3: Plasma cf-nDNA before and after physical stress induction 14

Supplementary Table 4: Plasma cf-mtDNA before and after psychosocial stress induction 16

Supplementary Table 5: Plasma cf-mtDNA before and after physical stress induction 18

Supplementary Table 6: Salivary cf-nDNA before and after psychosocial stress induction 20

Supplementary Table 7: Salivary cf-nDNA before and after physical stress induction 22

Supplementary Table 8: Salivary cf-mtDNA before and after psychosocial stress induction 24

Supplementary Table 9: Salivary cf-mtDNA before and after physical stress induction 26

**Supplementary Figures:**


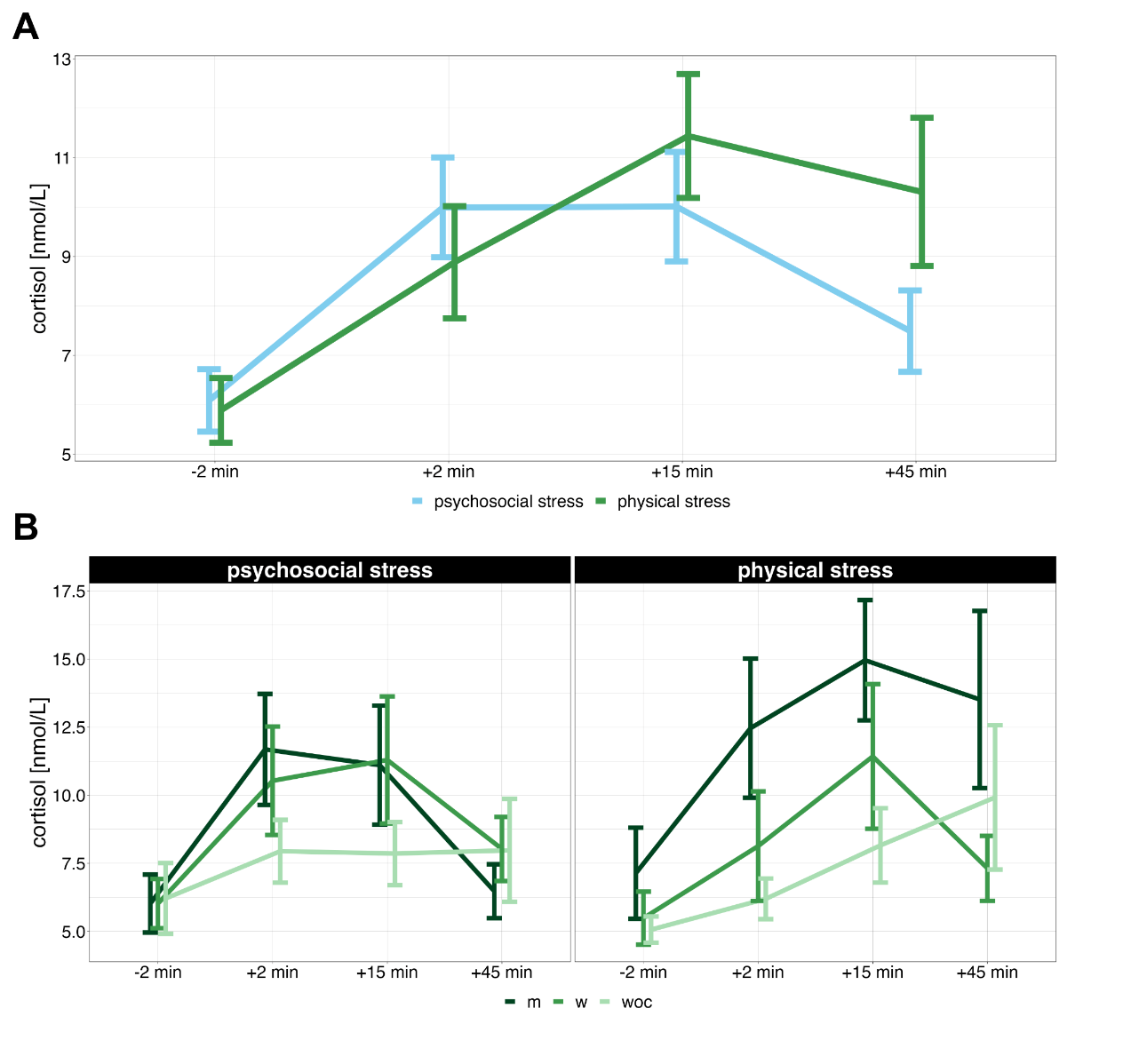


**Supplementary Figure 3: Salivary Cortisol Concentration**

This figure presents salivary cortisol concentrations measured before and after acute psychosocial and physical stress for all participants (A) and for the three groups (B; m = men, w = women, woc = women using oral hormonal contraception). Salivary cortisol concentrations are plotted in nmol/l over time. Error bars represent the standard error of the mean. The rmANOVA revealed that salivary cortisol levels differed significantly over the course of the experiment. Additionally, there was a two-way interaction between measurement time points and stress types. Follow-up analysis indicated significant differences in cortisol levels in the psychosocial and physical stress condition.


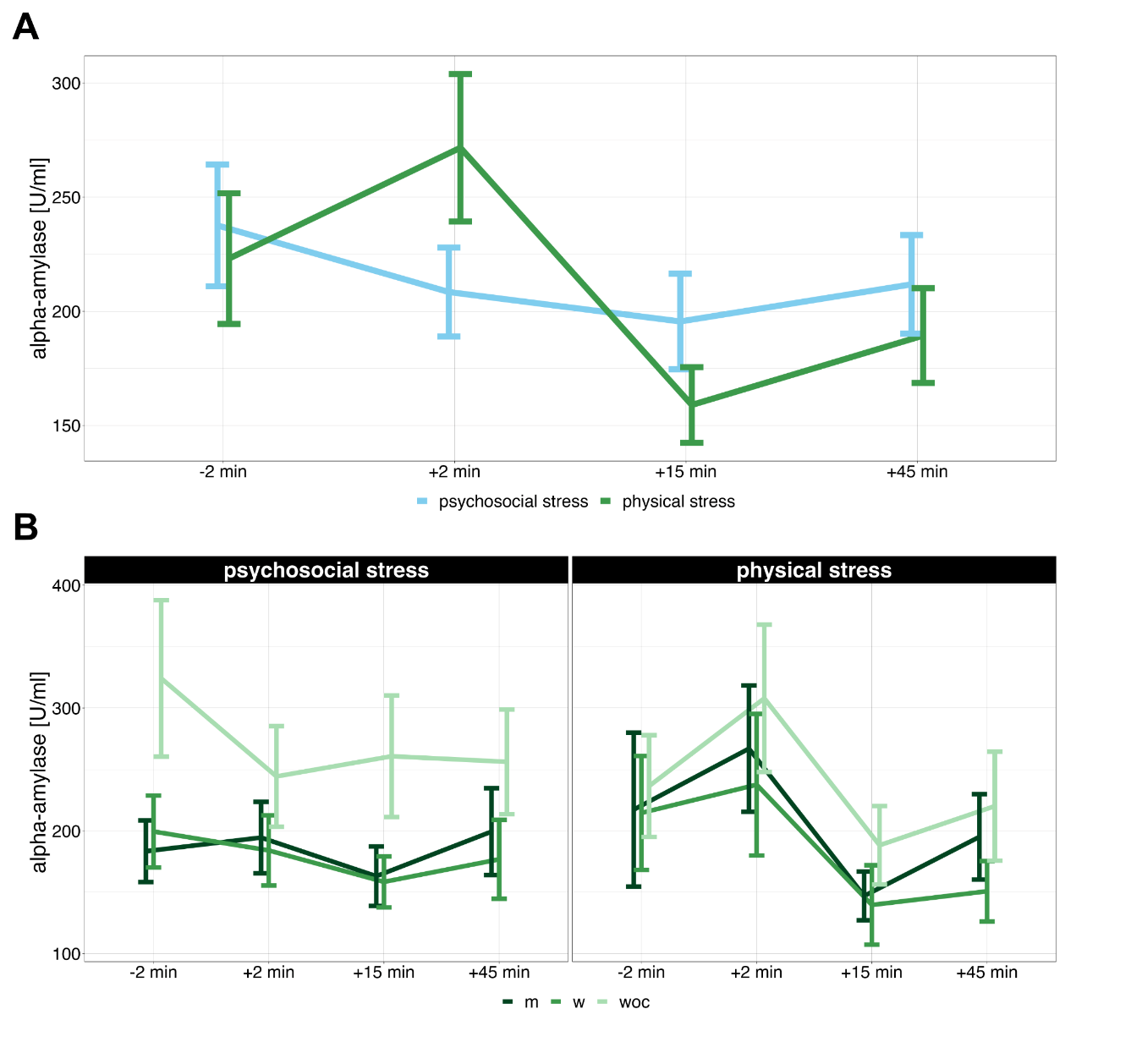


**Supplementary Figure 4: Salivary α-amylase Activity** **Levels**

This figure illustrates salivary α-amylase activity measured before and after acute psychosocial and physical stress for all participants (A) and for the three different groups (B; m = men, w = women, woc = women using oral hormonal contraception). Salivary α-amylase activity is plotted in U/ml over time. Error bars indicate the standard error of the mean. The rmANOVA for salivary α-amylase levels revealed a main effect for changes over time and an interaction between stress types and measurement time points. Post-hoc tests exhibit significant differences in both stress conditions.


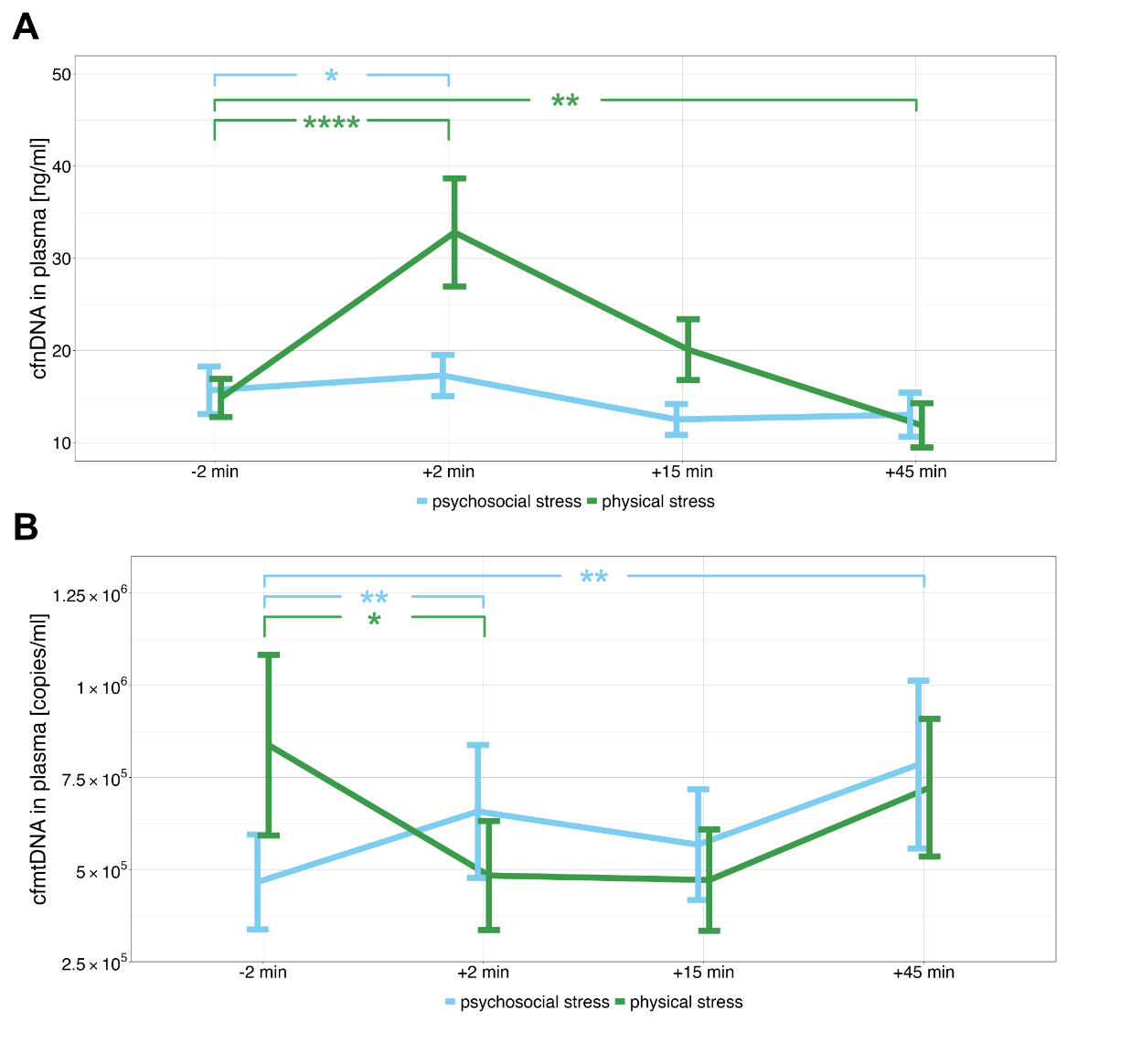


**Supplementary Figure 5: Plasma Levels of cf-nDNA and cf-mtDNA**

Plasma cf-nDNA concentration is presented in ng/ml over time, while cf-mtDNA concentration is represented in copies/ml over time (given that the weight of a haploid human genome is estimated to be approximately 3.23 pg, a concentration of 1 ng cf-nDNA/ml corresponds to approximately 310 genomic copies/ml).

A) Concentration of cf-nDNA in plasma before and after psychosocial and physical stress.

B) Concentration of cf-mtDNA in plasma before and after psychosocial and physical stress. Error bars represent the standard error of the mean. Follow-up analysis revealed significant differences in both the psychosocial and physical stress condition for plasma cf-nDNA and cf-mtDNA levels. * p < .05, ** p < .01, *** p < .001, **** p < .0001


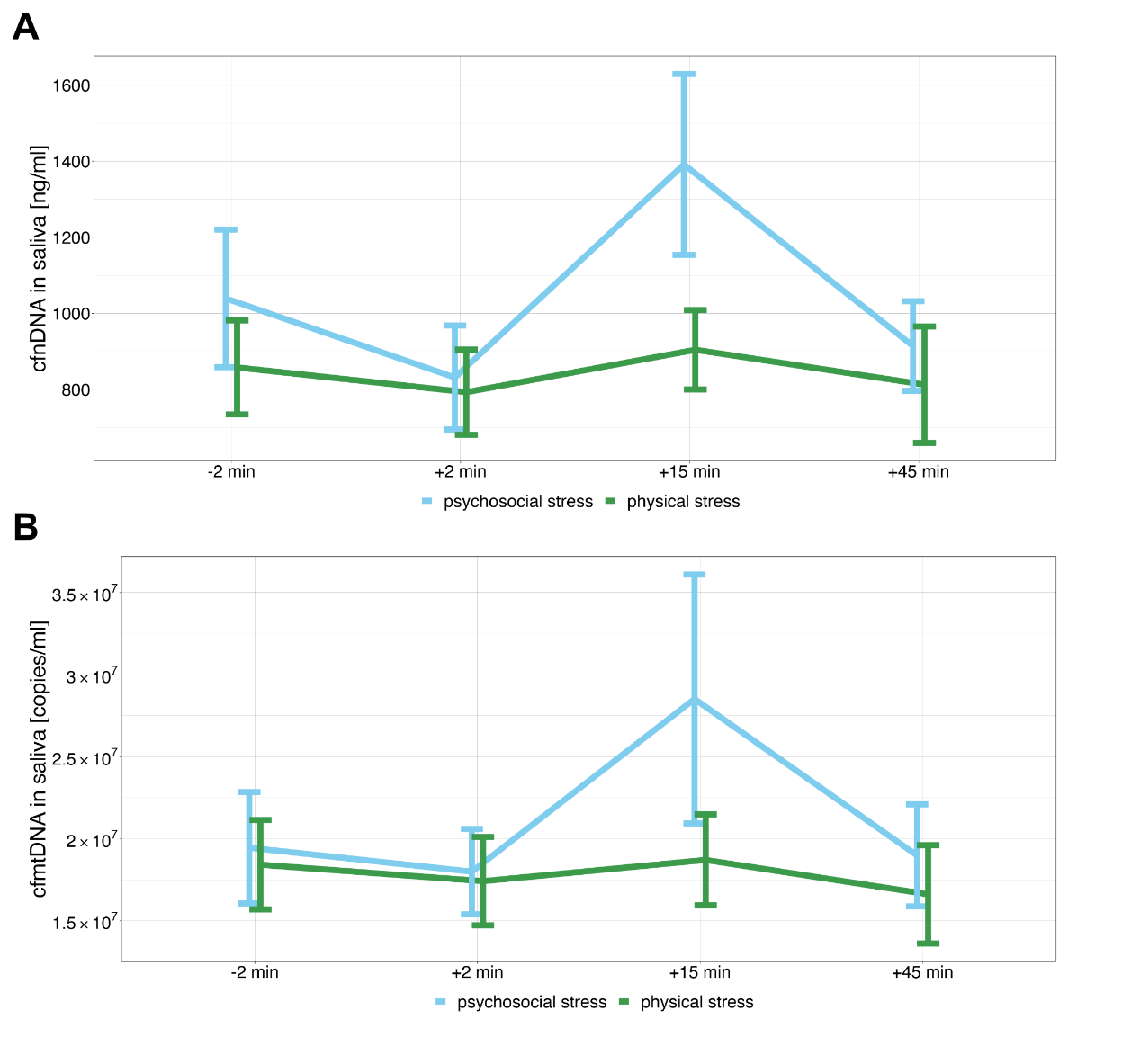


**Supplementary Figure 4: Quantification of cf-nDNA and cf-mtDNA in Saliva**

The concentration of cf-nDNA in saliva is plotted in ng/ml over time, while cf-mtDNA concentration is represented in copies/ml over time (given that the weight of a haploid human genome is estimated to be approximately 3.23 pg, a concentration of 1 ng cf-nDNA/ml corresponds to approximately 310 genomic copies/ml).

A) Cf-nDNA concentration in saliva before and after psychosocial and physical stress.

B) Cf-mtDNA concentration in saliva before and after psychosocial and physical stress. Error bars indicate the standard error of the mean. The rmANOVA of salivary cf-nDNA levels exhibited an unspecific main effect for changes over time. However, no other effects were significant.
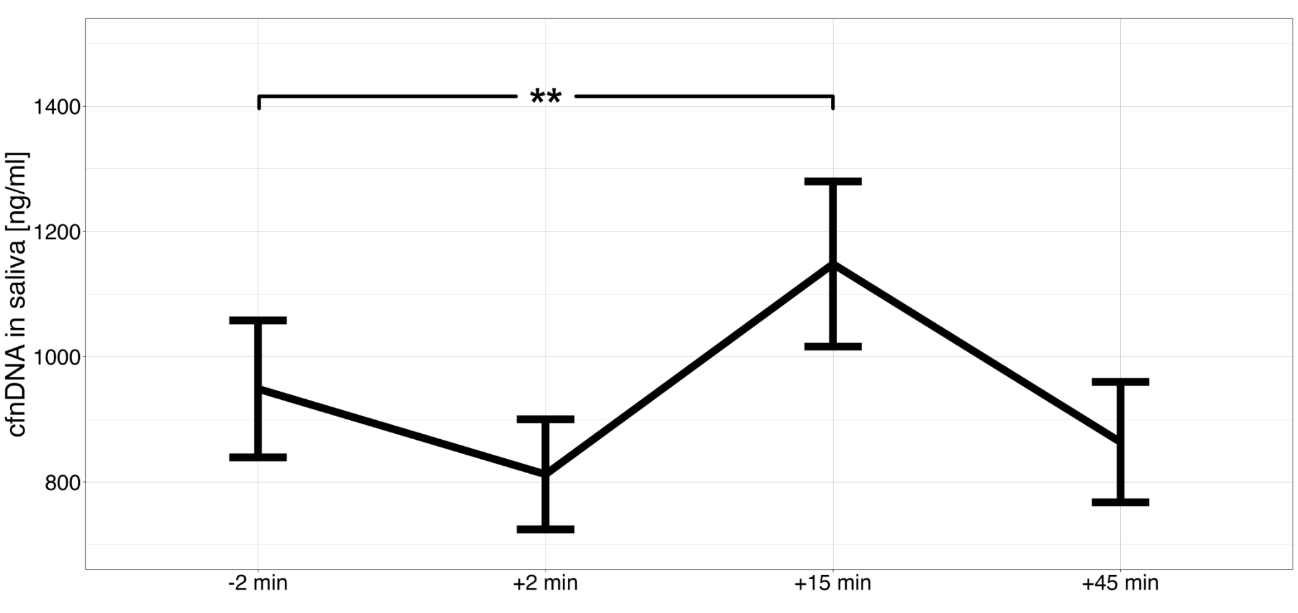


**Supplementary Figure 5: Quantification of cf-nDNA in Saliva**

The concentration of cf-nDNA in saliva is plotted in ng/ml over time, while cf-mtDNA concentration is represented in copies/ml over time (given that the weight of a haploid human genome is estimated to be approximately 3.23 pg, a concentration of 1 ng cfDNA/ml corresponds to approximately 310 genomic copies/ml). * p < .05, ** p < .01, *** p < .001, **** p < .0001

**SERS Results**

**Changes in subjective stress perception**

For the investigation of changes in subjective stress perception, data from 43 of the 46 participants were included in the analysis (13 men, 14 women, 16 women with oral hormonal contraception). Descriptive analysis of the data revealed that both “tense arousal” and “self-directed emotions” increased during both stress conditions. Additionally, these dimensions exhibited a peak at T2, followed by a subsequent decline. In contrast, the third dimension, “anxiety,” showed a more gradual progression over time, lacking a distinct peak following the stressor (see supplementary figure 6).

**Tense arousal**

The ANOVA for “tense arousal” revealed a significant main effect for the measuring timepoint (*F*(2, 42) = 50.52, *p* < .001, η^2^_p_ = 0.56) and an interaction between measuring timepoint and type of stressor (*F*(2, 15) = 5.87, *p* = .003, η^2^_p_ = 0.13). All other effects were not significant. Post hoc tests indicated a two-fold interaction effect within the types of stress, with significant differences in tense arousal between measuring timepoints for both the psychosocial (*F*(2, 16) = 35.70, *p* < .001, η^2^_p_ = 0.46) and the physical stressor (*F*(2, 42) = 27.00, *p* < .001, η^2^_p_ = 0.13). Additional pairwise comparisons (see supplemental figure 7) revealed the following:

**Psychosocial Stressor**

- Tense arousal was significantly higher at T1 compared to both T3 (*t*(42) = 4.40, *p* < .001) and T4 (*t*(42) = 7.35, *p* < .001).
- Tense arousal was significantly lower at T1 compared to T2 (*t*(42) = -3.10, *p* = .003).

**Physical Stressor**

- At T1, tense arousal was significantly higher after the stressor at T2 (*t*(42) = -2.84, *p* = .007).
- At T4, tense arousal was significantly lower compared to baseline levels (*t*(42) = 5.72, *p* < .001).
- Arousal levels at T3 did not differ significantly from baseline.

**Anxiety and self-directed emotions**

The data from the Anxiety and Self-Directed Emotions Scale were not statistically analyzed due to significant violations of the assumption of normality.


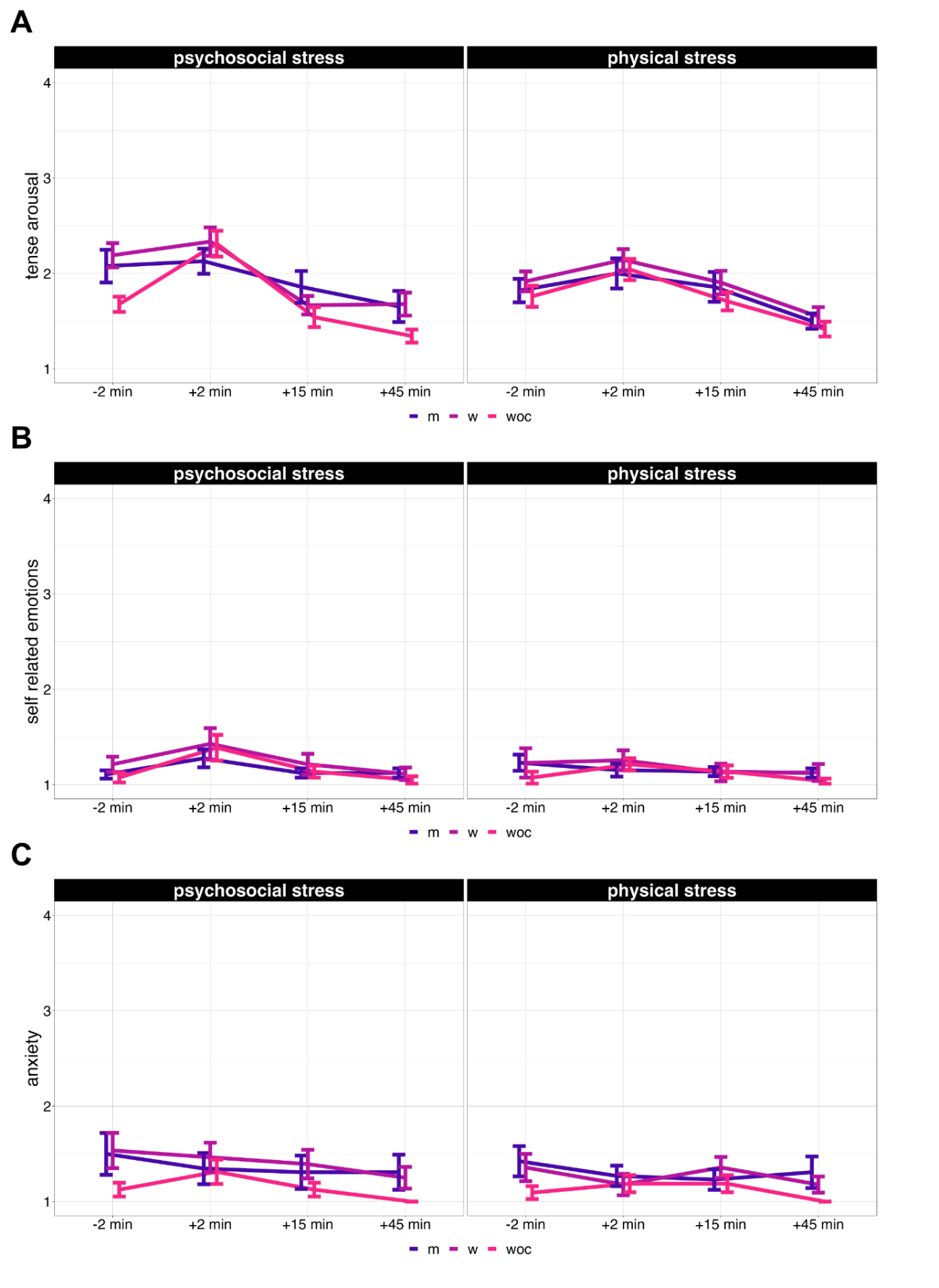


**Supplementary Figure 6: Summary of SERS Questionnaire Responses**

Trend analysis of A) tense arousal, B) self-related emotions, and C) anxiety responses across three groups (men [m], women [w], women with oral hormonal contraception [woc]) before and after psychosocial and physical stress.


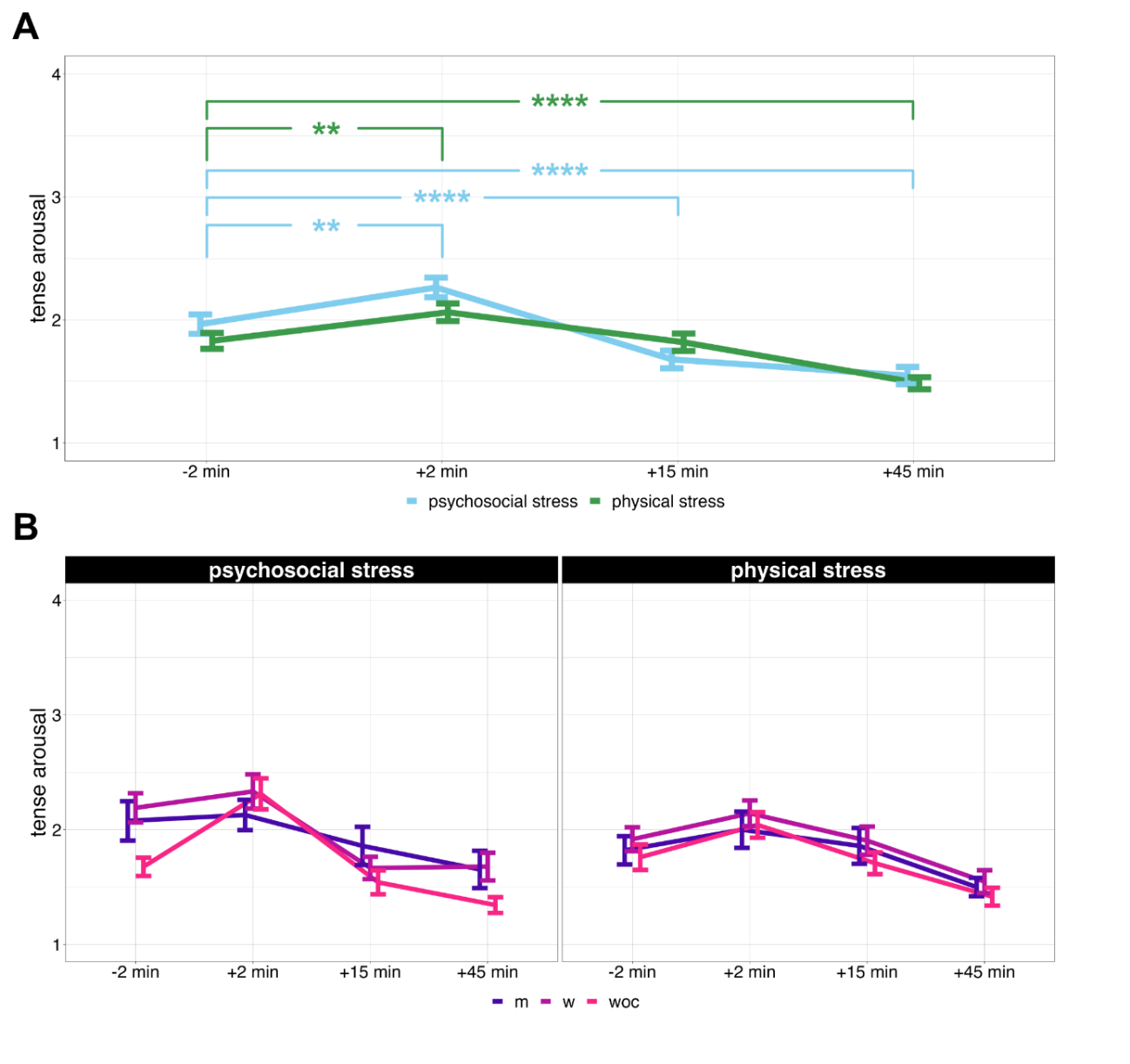


**Supplementary Figure 7: Distribution of Tense Arousal Responses in SERS**

A) Trend of tense arousal after acute psychosocial and physical stress for all participants. B) Trend of tense arousal for the three groups (men [m], women [w], women with oral hormonal contraception [woc]). Error bars represent the standard error of the mean. * p < .05, ** p < .01, *** p < .001, **** p < .0001


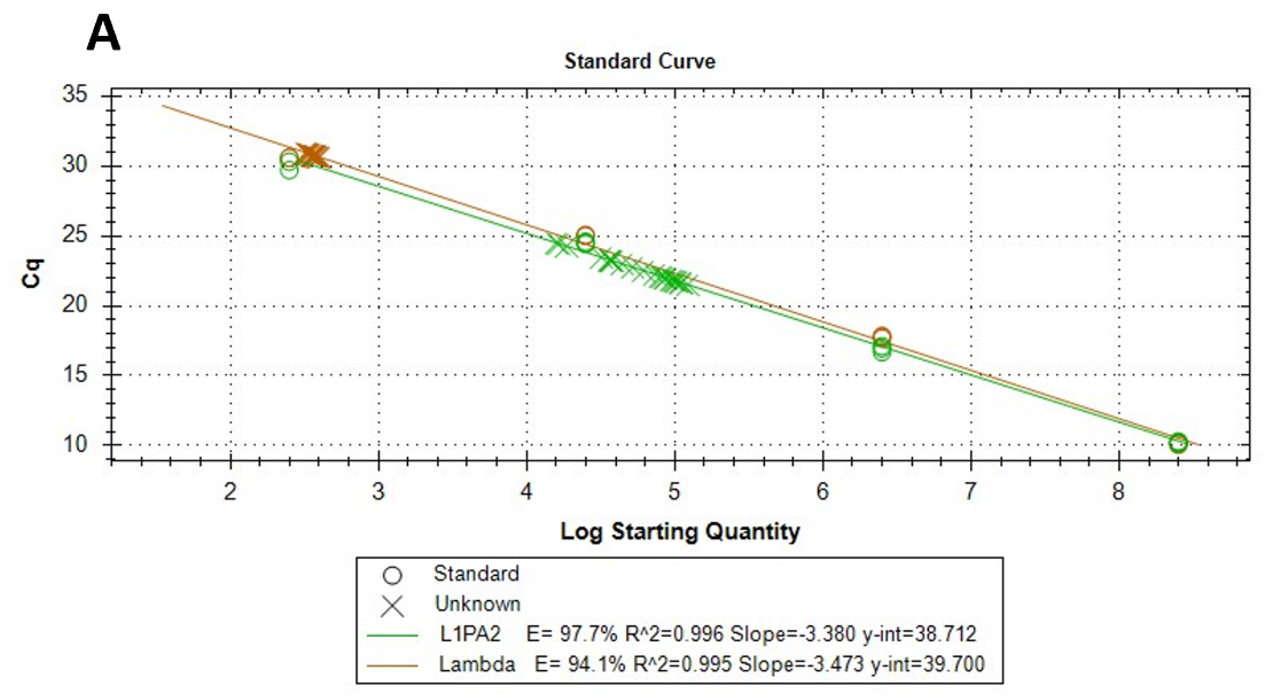


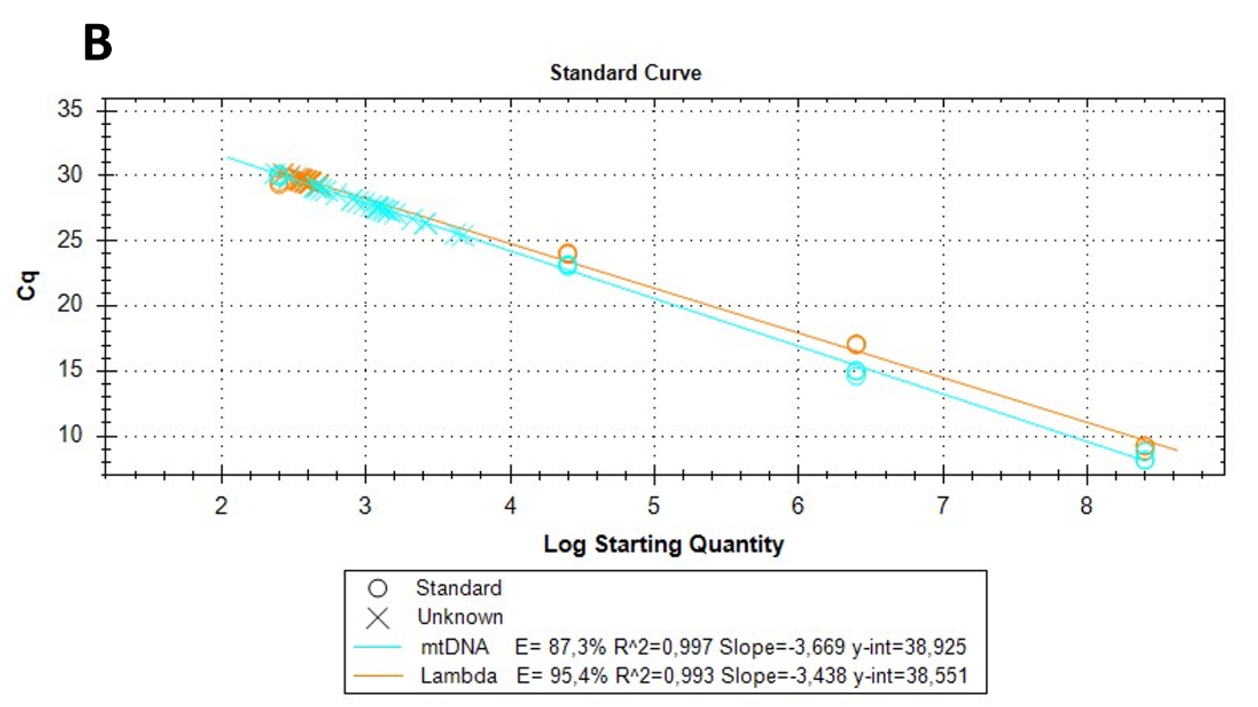


**Supplementary Figure 8: Standard curves for L1PA2 (cf-nDNA), mtDNA (cf-mtDNA), and Lambda-DNA BstEII 229 bp fragment (spike-in)**

The figures illustrate the standard curves for the two qPCR assays employed in cfDNA analysis. The x-axis represents the logarithm of the starting quantity, the y-axis the quantification cycle (Cq). All standard curves were generated using standard pools at four distinct concentrations: 2.5 x 108, 2.5 x 106, 2.5 x 104, and 2.5 x 102 copies/µl (refer to the “Material and Methods” section in the main text). A) Standard curves for the cf-nDNA target L1PA2 and the spike-in control (Lambda-DNA BstEII 229 bp fragment). B) Standard curves for the cf-mtDNA target (mtDNA) and the spike-in control (Lambda-DNA BstEII 229 bp fragment). Further information on primers, probes, standards and the genomic location of the amplification products can be found in Supplementary Table 1.

**Supplementary Tables:**

**Supplementary Table 1: Primers, probes, and standards**

| Target Name | Binding Site Position | Sequence (5´- 3´) |
| --- | --- | --- |
| L1PA2 forward (300nM) | % | AATGCCGCAATAAACATAC |
| L1PA2 reverse (300nM) | % | GAACTAGAGATACCATTTGACC |
| FAM-L1PA2-MGBEQ Probe (250nM) | % | [FAM] TGC ATG TGT CTT TAT AGC AGC ATG A [MGBEQ] |
| L1PA2 standard  114 bp | % | ATAATGCCGCAATAAACATACGTGTGCATGTGTCTTTATAGCA  GCATGATTTATAGTCATTTGGGTATATACCCAGTAATGGGATG  GCTGGGTCAAATGTATCTCTAGTTCTA |
|  |  |  |
| mtDNA forward (300nM) | mt 16 528 - 16 548 | CTAAATAGCCCACACGTTCCC |
| mtDNA reverse (300nM) | mt 23 - 42 | AGAGCTCCCGTGAGTGGTTA |
| HEX-mtDNA-BHQ1 Probe (250nM) | mt 16 560 - 16 510 | [HEX]CAT[+C]AC[+G]AT[+G][+G]A[+T]CA[+C]AGGT[BHQ1] |
| mtDNA standard  84 bp | mt 16 528 - 42 | CTAAATAGCCCACACGTTCCCCTTAAATAAGACATCACGATGG  ATCACAGGTCTATCACCCTATTAACCACTCACGGGAGCTCT |
|  |  |  |
| Lambda forward (300nM) | λ 13 436- 13 455 | ATATCCGGCAGGAAACACTG |
| Lambda reverse (300nM) | λ 13 535- 13 516 | TAACGTTCTCCACCGACCTC |
| Cy5-Lambda-BHQ3 Probe (250nM) | λ 13 457- 13 476 | [Cy5]ATGAATGCACCCGTGCGGAG[BHQ3] |
| Lambda BstEII 229 bp fragment  [NC_049951.1](https://www.ncbi.nlm.nih.gov/nucleotide/NC_049951.1?report=genbank&log$=nuclalign&blast_rank=1&RID=9ZH686ZY013) | λ 13 349 – 13 577 | GTGACCTGCGCAAAATGGTCGTCGCGGGTCAGTATGCTGCGTG  TTGAGTTCAGCGCAGAGTTTGAACAGGTGGTGAACTGATGCAG  GATATCCGGCAGGAAACACTGAATGAATGCACCCGTGCGGAGC  AGTCGGCCAGCGTGGTGCTCTGGGAAATCGACCTGACAGAGGT  CGGTGGAGAACGTTATTTTTTCTGTAATGAGCAGAACGAAAAA  GGTGAGCCGGTCAC |

This table provides the sequences of the primers (300 nM), probes (250 nM) and standards used for cfDNA qPCR targeting three specific elements: L1PA2 (cf-nDNA), mtDNA (cf-mtDNA), and Lambda DNA BstEII 229 bp fragment (spike-in). The cf-nDNA target focuses on the L1PA2 elements a subfamily of the LINE1 transposable elements. Due to the abundant presence of these transposable DNA elements in the human genome, the DNA sequence is present approximately 2,374 times when using the depicted primers and probe. Abbreviations used are as follows: BHQ = Black Hole Quencher®; [+] = Locked Nucleic Acids (LNA); MGBEQ = Minor Groove Binder Eclipse Quencher

**Supplementary Table 2: Plasma cf-nDNA before and after psychosocial stress induction**

| psychosocial stress | | plasma cf-nDNA [ng/ml] | | | | |
| --- | --- | --- | --- | --- | --- | --- |
| participant | **group** | **T1** | | **T2** | **T3** | **T4** |
| 1 | m | 4.70 | | 7.00 | 4.04 | 4.18 |
| 2 | woc | 11.24 | | 9.70 | 2.13 | 3.73 |
| 3 | m | 14.51 | | 14.27 | 16.53 | 12.11 |
| 4 | m | 4.11 | | 7.68 | 5.16 | 1.60 |
| 5 | w | 82.63 | | 7.70 | 3.89 | 23.72 |
| 6 | m | 4.24 | | 3.02 | 1.63 | 4.47 |
| 7 | m | 3.42 | | 1.80 | 25.53 | 5.50 |
| 8 | w | 2.29 | | 4.27 | 2.06 | 0.42 |
| 11 | woc | 1.60 | | 4.44 | 1.57 | 7.37 |
| 12 | woc | 1.95 | | 1.49 | 1.67 | 4.51 |
| 13 | m | 3.40 | | 5.46 | 4.90 | 3.86 |
| 14 | woc | 1.38 | | 4.72 | 1.90 | 1.52 |
| 15 | woc | 2.59 | | 2.77 | 1.69 | 2.29 |
| 16 | woc | 4.56 | | 26.95 | 39.14 | 6.24 |
| 17 | woc | 17.47 | | 9.37 | 20.62 | 15.32 |
| 18 | m | 7.09 | | 7.87 | 5.36 | 3.97 |
| 19 | w | 8.02 | | 10.00 | 4.32 | 6.12 |
| 20 | m | 4.56 | | 10.36 | 4.74 | 5.56 |
| 21 | m | 13.01 | | 12.92 | 11.06 | 6.44 |
| 22 | w | 12.06 | | 19.83 | 9.47 | 7.00 |
| 23 | woc | 15.73 | | 15.47 | 8.36 | 6.43 |
| 24 | m | 13.28 | | 38.43 | 14.54 | 84.42 |
| 25 | m | 18.23 | | 17.07 | 14.32 | 10.55 |
| 26 | woc | 14.27 | | 29.99 | 18.02 | 16.16 |
| 27 | m | 56.02 | | 40.26 | 24.43 | 20.29 |
| 28 | woc | 56.82 | | 44.92 | 54.43 | 68.05 |
| 29 | w | 12.09 | | 13.86 | 8.59 | 6.00 |
| 30 | w | 17.19 | | 19.12 | 11.86 | 11.99 |
| 31 | w | 14.12 | | 18.30 | 13.24 | 23.32 |
| 32 | m | 17.96 | | 24.08 | 21.75 | 16.11 |
| 34 | woc | 60.21 | | 83.22 | 21.93 | 29.67 |
| 35 | m | 35.72 | | 25.38 | 20.03 | 20.39 |
| 36 | woc | 20.57 | | 14.95 | 15.22 | 9.07 |
| 37 | woc | 9.65 | | 32.10 | 38.90 | 18.73 |
|  |  |  | |  |  |  |
| psychosocial stress | | | **plasma cf-nDNA [ng/ml]** | | | |
| participant | **group** | **T1** | | **T2** | **T3** | **T4** |
| 38 | w | 17.88 | | 35.11 | 18.24 | 29.24 |
| 39 | m | 9.94 | | 15.40 | 6.07 | 7.08 |
| 40 | woc | 14.79 | | 11.39 | 6.66 | 9.78 |
| 41 | w | 7.75 | | 9.46 | 7.50 | 5.54 |
| 42 | w | 12.22 | | 14.49 | 8.01 | 6.82 |
| 43 | woc | 7.40 | | 7.74 | 8.64 | 5.02 |
| 44 | w | 17.50 | | 24.15 | 10.51 | 9.22 |
| 45 | w | 15.49 | | 16.43 | 10.73 | 6.43 |
| 46 | woc | 12.37 | | 25.27 | 12.75 | 13.03 |
| 47 | w | 8.27 | | 12.88 | 9.42 | 14.49 |
|  |  |  | |  |  |  |
| arithmetic mean | ng/ml | 15.69 | | 17.30 | 12.54 | 13.04 |
| SD |  | 16.91 | | 14.70 | 10.98 | 15.64 |

The table presents plasma cf-nDNA concentrations [ng/ml] before and after the onset of psychosocial stress. It includes participant numbers, participant groups (m = men, w = women, woc = women using oral hormonal contraception), and cf-nDNA amounts measured by qPCR at four time points: baseline (-2 min, T1), immediately after the stressor (+2 min, T2), at the expected plateau of cfDNA increase (+15 min, T3), and at the anticipated return to baseline (+45 min, T4). Given that the weight of a haploid human genome is approximately 3.23 pg, a concentration of 1 ng cf-nDNA/ml corresponds to about 310 genomic copies/ml.

**Supplementary Table 3: Plasma cf-nDNA before and after physical stress induction**

| physical stress | | plasma cf-nDNA [ng/ml] | | | |
| --- | --- | --- | --- | --- | --- |
| participant | **group** | **T1** | **T2** | **T3** | **T4** |
| 1 | **m** | **10.73** | **15.05** | **1.80** | **8.79** |
| 2 | woc | 4.74 | 1.36 | 35.56 | 3.24 |
| 3 | **m** | **19.84** | **49.56** | **19.72** | **27.06** |
| 4 | m | 5.41 | 6.53 | 4.55 | 1.73 |
| 5 | **w** | **10.71** | **26.79** | **3.71** | **46.67** |
| 6 | m | 18.09 | 18.16 | 2.50 | 1.90 |
| 7 | **m** | **6.20** | **3.74** | **4.75** | **1.58** |
| 8 | w | 0.87 | 5.20 | 7.09 | 1.87 |
| 11 | **woc** | **5.76** | **4.81** | **2.94** | **0.62** |
| 12 | woc | 4.11 | 2.66 | 1.17 | 1.64 |
| 13 | **m** | **4.03** | **11.32** | **10.52** | **6.42** |
| 14 | woc | 3.86 | 6.82 | 4.36 | 5.22 |
| 15 | **woc** | **5.84** | **8.56** | **3.79** | **1.51** |
| 16 | woc | 13.08 | 25.45 | 12.73 | 5.00 |
| 17 | **woc** | **11.76** | **21.20** | **10.28** | **4.90** |
| 18 | m | 4.71 | 7.04 | 5.95 | 1.03 |
| 19 | **w** | **10.31** | **4.47** | **4.77** | **2.64** |
| 20 | m | 7.49 | 35.69 | 18.83 | 7.13 |
| 21 | **m** | **5.77** | **28.91** | **16.34** | **8.79** |
| 22 | w | 11.68 | 19.04 | 5.72 | 5.46 |
| 23 | **woc** | **14.37** | **30.68** | **21.48** | **8.17** |
| 24 | m | 17.58 | 55.91 | 36.21 | 14.19 |
| 25 | **m** | **15.71** | **56.66** | **31.31** | **19.86** |
| 26 | woc | 15.40 | 21.30 | 14.06 | 7.50 |
| 27 | **m** | **53.94** | **234.16** | **112.42** | **19.50** |
| 28 | woc | 65.73 | 51.10 | 23.75 | 14.21 |
| 29 | **w** | **9.07** | **10.94** | **12.09** | **4.04** |
| 30 | w | 17.85 | 24.54 | 22.60 | 97.07 |
| 31 | **w** | **57.01** | **42.51** | **31.90** | **14.87** |
| 32 | m | 20.04 | 58.60 | 28.88 | 11.74 |
| 34 | **woc** | **26.57** | **44.34** | **20.34** | **10.09** |
| 35 | m | 29.37 | 102.00 | 39.15 | 16.75 |
| 36 | **woc** | **22.33** | **50.31** | **37.39** | **9.49** |

| physical stress | | plasma cf-nDNA [ng/ml] | | | |
| --- | --- | --- | --- | --- | --- |
| participant | **group** | **T1** | **T2** | **T3** | **T4** |
| 37 | woc | 8.57 | 8.39 | 4.63 | 12.26 |
| 38 | w | 5.77 | 22.06 | 15.80 | 6.63 |
| 39 | m | 10.79 | 107.15 | 96.34 | 32.31 |
| 40 | woc | 8.16 | 20.31 | 12.35 | 8.69 |
| 41 | w | 10.19 | 28.12 | 17.77 | 17.35 |
| 42 | w | 16.42 | 32.46 | 19.88 | 9.77 |
| 43 | woc | 16.25 | 25.00 | 11.55 | 3.57 |
| 44 | w | 13.36 | 23.99 | 31.23 | 12.04 |
| 45 | w | 21.33 | 45.62 | 38.59 | 17.59 |
| 46 | woc | 6.67 | 23.31 | 20.03 | 7.68 |
| 47 | w | 7.05 | 22.12 | 7.81 | 4.34 |
|  |  |  |  |  |  |
| arithmetic  mean | ng/ml | 14.88 | 32.82 | 20.11 | 11.89 |
| SD |  | 13.56 | 38.43 | 21.64 | 15.75 |

The table displays plasma cf-nDNA concentrations [ng/ml] before and after the physical stress condition. It includes participant numbers, participant groups (m = men, w = women, woc = women using oral hormonal contraception), and cf-nDNA amounts measured by qPCR at four time points: baseline (-2 min, T1), immediately after the stressor (+2 min, T2), at the expected plateau of cfDNA increase (+15 min, T3), and at the anticipated return to baseline (+45 min, T4). Given that the weight of a haploid human genome is approximately 3.23 pg, a concentration of 1 ng cf-nDNA/ml corresponds to about 310 genomic copies/ml.

**Supplementary Table 4: Plasma cf-mtDNA before and after psychosocial stress induction**

| psychosocial stress | | plasma cf-mtDNA [copies/ml] | | | |
| --- | --- | --- | --- | --- | --- |
| participant | **group** | **T1** | **T2** | **T3** | **T4** |
| 1 | m | 446 337 | 739 134 | 48 804 | 563 498 |
| 2 | woc | 67 328 | 346 611 | 184 515 | 27 512 |
| 3 | m | 101 315 | 360 101 | 85 484 | 242 242 |
| 4 | m | 121 798 | 243 333 | 228 708 | 113 102 |
| 5 | w | 345 638 | 186 111 | 497 445 | 144 175 |
| 6 | m | 112 736 | 39 449 | 868 197 | 79 268 |
| 8 | w | 188 512 | 169 857 | 141 040 | 985 642 |
| 11 | woc | 48 175 | 338 669 | 104 875 | 242 712 |
| 13 | m | 224 645 | 267 570 | 432 021 | 348 841 |
| 14 | woc | 62 802 | 252 044 | 59 832 | 52 648 |
| 15 | woc | 305 565 | 366 610 | 214 679 | 263 880 |
| 16 | woc | 259 560 | 271 107 | 328 396 | 291 510 |
| 17 | woc | 204 856 | 189 620 | 315 380 | 857 999 |
| 18 | m | 625 163 | 185 754 | 340 401 | 440 667 |
| 19 | w | 195 903 | 317 617 | 63 712 | 156 688 |
| 20 | m | 120 844 | 139 359 | 170 453 | 237 070 |
| 21 | m | 157 834 | 150 969 | 138 221 | 119 625 |
| 22 | w | 39 734 | 200 722 | 39 848 | 327 869 |
| 23 | woc | 104 778 | 597 308 | 113 137 | 111 118 |
| 24 | m | 116 565 | 482 781 | 105 505 | 431 206 |
| 25 | m | 163 371 | 387 730 | 219 002 | 177 608 |
| 26 | woc | 548 135 | 534 652 | 687 487 | 693 048 |
| 27 | m | 437 935 | 897 234 | 299 475 | 907 794 |
| 28 | woc | 1 361 966 | 823 252 | 1 114 272 | 8 397 775 |
| 29 | w | 392 851 | 587 753 | 1 081 497 | 489 225 |
| 30 | w | 318 185 | 518 168 | 391 432 | 517 806 |
| 31 | w | 270 071 | 612 576 | 367 341 | 1 519 870 |
| 32 | m | 333 605 | 493 571 | 1 362 829 | 1 105 892 |
| 34 | woc | 1 355 792 | 1 619 575 | 395 962 | 1 740 677 |
| 35 | m | 1 403 959 | 1 006 546 | 2 245 727 | 1 575 412 |
| 36 | woc | 1 590 586 | 1 127 384 | 2 834 220 | 688 609 |
| 37 | woc | 500 680 | 506 962 | 128 645 | 396 870 |
|  | |  | | | |
| psychosocial stress | | **plasma cf-mtDNA [copies/ml]** | | | |
| participant | **group** | **T1** | **T2** | **T3** | **T4** |
| 38 | w | 326 023 | 4 915 524 | 1 018 350 | 1 230 343 |
| 39 | m | 79 087 | 156 496 | 176 132 | 273 207 |
| 40 | woc | 318 492 | 336 218 | 194 166 | 229 538 |
| 41 | w | 184 059 | 206 384 | 159 466 | 304 151 |
| 42 | w | 215 392 | 163 493 | 35 682 | 26 849 |
| 43 | woc | 139 030 | 240 898 | 318 508 | 440 764 |
| 44 | w | 30 413 | 65 551 | 330 161 | 558 453 |
| 45 | w | 559 390 | 301 381 | 297 852 | 233 755 |
| 46 | woc | 5 142 536 | 6 251 505 | 5 559 890 | 5 257 351 |
| 47 | w | 78 583 | 38 643 | 148 734 | 174 172 |
|  |  |  |  |  |  |
| arithmetic mean | copies/ml | 466 672 | 658 005 | 567 797 | 785 153 |
| SD |  | 823 565 | 1 154 269 | 963 715 | 1 457 115 |

The table presents plasma cf-mtDNA concentrations [copies/ml] before and after the psychosocial stress condition. It includes participant numbers, participant groups (m = men, w = women, woc = women using oral hormonal contraception), and cf-mtDNA amounts measured by qPCR at four time points: baseline (-2 min, T1), immediately after the stressor (+2 min, T2), at the expected plateau of cfDNA increase (+15 min, T3), and at the anticipated return to baseline (+45 min, T4).

**Supplementary Table 5: Plasma cf-mtDNA before and after physical stress induction**

| physical stress | | plasma cf-mtDNA [copies/ml] | | | |
| --- | --- | --- | --- | --- | --- |
| participant | **group** | **T1** | **T2** | **T3** | **T4** |
| 1 | m | 137 606 | 150 383 | 531 311 | 777 063 |
| 2 | woc | 22 932 | 26 087 | 184 634 | 56 191 |
| 3 | m | 335 397 | 70 816 | 87 060 | 116 602 |
| 4 | m | 80 206 | 150 468 | 257 128 | 140 153 |
| 5 | w | 375 030 | 33 852 | 437 401 | 534 782 |
| 6 | m | 106 998 | 45 173 | 51 084 | 174 877 |
| 8 | w | 2 946 024 | 253 178 | 210 016 | 180 270 |
| 11 | woc | 172 234 | 111 191 | 451 222 | 167 850 |
| 13 | m | 261 072 | 316 303 | 216 504 | 553 422 |
| 14 | woc | 107 733 | 281 322 | 89 495 | 332 652 |
| 15 | woc | 683 048 | 189 063 | 301 657 | 223 041 |
| 16 | woc | 311 835 | 327 111 | 264 067 | 438 281 |
| 17 | woc | 37 128 | 70 589 | 129 544 | 255 641 |
| 18 | m | 336 527 | 153 284 | 260 912 | 281 835 |
| 19 | w | 460 120 | 81 763 | 188 351 | 124 266 |
| 20 | m | 474 614 | 209 150 | 120 629 | 752 775 |
| 21 | m | 108 304 | 96 763 | 100 182 | 100 845 |
| 22 | w | 126 860 | 89 715 | 280 356 | 179 432 |
| 23 | woc | 210 466 | 101 220 | 97 706 | 257 065 |
| 24 | m | 278 124 | 281 886 | 330 451 | 264 373 |
| 25 | m | 181 520 | 155 142 | 121 983 | 539 467 |
| 26 | woc | 415 128 | 928 816 | 444 867 | 1 183 711 |
| 27 | m | 452 463 | 878 426 | 391 048 | 937 286 |
| 28 | woc | 919 802 | 576 292 | 193 923 | 707 771 |
| 29 | w | 1 046 329 | 479 606 | 545 643 | 316 309 |
| 30 | w | 2 281 404 | 94 306 | 180 826 | 1 911 937 |
| 31 | w | 2 178 215 | 160 367 | 271 604 | 334 196 |
| 32 | m | 859 494 | 5 351 635 | 1 988 802 | 615 726 |
| 34 | woc | 417 085 | 486 052 | 685 855 | 348 243 |
| 35 | m | 9 187 018 | 1 752 828 | 2 830 134 | 673 783 |
| 36 | woc | 3 725 672 | 849 890 | 836 571 | 2 713 093 |
|  |  |  |  |  |  |
| physical stress | | **plasma cf-mtDNA [copies/ml]** | | | |
| participant | **group** | **T1** | **T2** | **T3** | **T4** |
| 37 | woc | 864 192 | 599 994 | 211 974 | 5 748 001 |
| 38 | w | 78 880 | 25 445 | 111 764 | 147 672 |
| 39 | m | 142 597 | 310 511 | 225 938 | 990 082 |
| 40 | woc | 455 684 | 196 647 | 269 355 | 427 571 |
| 41 | w | 158 407 | 137 105 | 176 993 | 445 504 |
| 42 | w | 70 199 | 217 413 | 169 207 | 111 718 |
| 43 | woc | 289 666 | 118 948 | 229 714 | 186 194 |
| 44 | w | 580 700 | 38 754 | 24 877 | 398 722 |
| 45 | w | 175 163 | 126 267 | 105 217 | 167 173 |
| 46 | woc | 3 094 678 | 3 335 302 | 5 125 364 | 5 433 243 |
| 47 | w | 46 014 | 476 888 | 87 672 | 99 982 |
|  |  |  |  |  |  |
| arithmetic mean | copies/ml | 837 918 | 484 189 | 471 882 | 722 591 |
| SD |  | 1 568 987 | 946 966 | 880 511 | 1 196 154 |

The table presents plasma cf-mtDNA concentrations [copies/ml] before and after the physical stress condition. It includes participant numbers, participant groups (m = men, w = women, woc = women using oral hormonal contraception), and cf-mtDNA amounts measured by qPCR at four time points: baseline (-2 min, T1), immediately after the stressor (+2 min, T2), at the expected plateau of cfDNA increase (+15 min, T3), and at the anticipated return to baseline (+45 min, T4).

**Supplementary Table 6: Salivary cf-nDNA before and after psychosocial stress induction**

| psychosocial stress | | salivary cf-nDNA [ng/ml] | | | |
| --- | --- | --- | --- | --- | --- |
| participant | **group** | **T1** | **T2** | **T3** | **T4** |
| 4 | m | 4 216.33 | 3 497.21 | 489.65 | 702.27 |
| 5 | w | 1 568.64 | 329.23 | 4 324.68 | 1 939.12 |
| 6 | m | 1 511.66 | 890.14 | 2 115.88 | 2 147.98 |
| 7 | m | 4 117.47 | 2 864.83 | 1 856.08 | 1 483.00 |
| 8 | w | 468.98 | 270.89 | 353.70 | 1 120.87 |
| 9 | w | 230.55 | 412.69 | 649.67 | 223.63 |
| 10 | w | 703.01 | 373.39 | 419.32 | 322.71 |
| 11 | woc | 2 097.50 | 1 784.37 | 2 421.37 | 1 637.45 |
| 12 | woc | 428.74 | 215.17 | 426.15 | 241.65 |
| 13 | m | 2 038.42 | 1 471.56 | 3 040.20 | 2 448.12 |
| 14 | woc | 1 261.52 | 1 775.70 | 743.28 | 1 201.75 |
| 15 | woc | 889.37 | 736.92 | 1 841.70 | 997.85 |
| 16 | woc | 191.69 | 205.81 | 71.50 | 33.75 |
| 17 | woc | 902.05 | 895.59 | 888.12 | 876.93 |
| 18 | m | 1 358.37 | 1 208.04 | 1 505.67 | 901.65 |
| 19 | w | 1 818.45 | 932.70 | 298.45 | 2 178.48 |
| 20 | m | 25.84 | 52.56 | 44.92 | 27.21 |
| 21 | m | 1 337.70 | 294.63 | 2 371.84 | 1 379.26 |
| 22 | w | 1 492.47 | 740.36 | 3 055.85 | 860.36 |
| 23 | woc | 4 668.86 | 3 116.24 | 1 367.37 | 3 184.26 |
| 24 | m | 240.79 | 172.62 | 195.53 | 283.31 |
| 25 | m | 439.44 | 549.42 | 578.64 | 652.10 |
| 26 | woc | 970.71 | 695.79 | 2 436.23 | 419.84 |
| 27 | m | 457.02 | 370.40 | 360.93 | 295.49 |
| 28 | woc | 641.45 | 387.86 | 566.07 | 220.00 |
| 29 | w | 3 371.26 | 1 689.61 | 1 745.41 | 1 495.81 |
| 30 | w | 464.28 | 262.73 | 534.90 | 337.23 |
| 31 | w | 80.82 | 80.23 | 569.45 | 1 264.68 |
| 32 | m | 513.28 | 468.77 | 390.10 | 975.72 |
| 34 | woc | 183.20 | 232.56 | 544.55 | 464.43 |
| 35 | m | 124.99 | 142.47 | 221.60 | 491.09 |
|  |  |  |  |  |  |
| psychosocial stress | | **salivary cf-nDNA [ng/ml]** | | | |
| participant | **group** | **T1** | **T2** | **T3** | **T4** |
| 37 | woc | 18.87 | 2 860.18 | 6 977.78 | 620.17 |
| 38 | w | 223.29 | 155.05 | 516.51 | 181.37 |
| 39 | m | 532.31 | 954.59 | 5 217.01 | 1 943.75 |
| 40 | woc | 170.35 | 400.08 | 4 384.57 | 1 921.02 |
| 41 | w | 1 162.32 | 936.87 | 1 551.85 | 936.28 |
| 42 | w | 318.87 | 165.06 | 141.92 | 88.44 |
| 43 | woc | 50.23 | 38.22 | 71.89 | 37.75 |
| 44 | w | 263.37 | 437.77 | 361.34 | 369.50 |
| 45 | w | 1 646.83 | 320.76 | 1 189.77 | 1 028.75 |
| 46 | woc | 372.43 | 1 406.43 | 1 158.11 | 351.80 |
| 47 | w | 82.04 | 131.56 | 445.86 | 120.26 |
|  |  |  |  |  |  |
| arithmetic  mean | ng/ml | 1 039.42 | 831.60 | 1 391.56 | 914.46 |
| SD |  | 1 157.43 | 874.11 | 1 522.11 | 754.70 |

The table presents salivary cf-nDNA concentrations [ng/ml] before and after the psychosocial stress condition. It includes participant numbers, participant groups (m = men, w = women, woc = women using oral hormonal contraception), and cf-nDNA amounts measured by qPCR at four time points: baseline (-2 min, T1), immediately after the stressor (+2 min, T2), at the expected plateau of cfDNA increase (+15 min, T3), and at the anticipated return to baseline (+45 min, T4). Given that the weight of a haploid human genome is approximately 3.23 pg, a concentration of 1 ng cf-nDNA/ml corresponds to about 310 genomic copies/ml.

**Supplementary Table 7: Salivary cf-nDNA before and after physical stress induction**

| physical stress | | salivary cf-nDNA [ng/ml] | | | |
| --- | --- | --- | --- | --- | --- |
| participant | **group** | **T1** | **T2** | **T3** | **T4** |
| 4 | m | 2 173.23 | 1 121.55 | 1 192.83 | 768.17 |
| 5 | w | 1 033.02 | 2 019.34 | 654.66 | 968.07 |
| 6 | m | 1 263.58 | 1 559.85 | 1 967.23 | 1 562.67 |
| 7 | m | 1 878.07 | 2 401.07 | 1 779.99 | 5 215.63 |
| 8 | w | 848.93 | 799.80 | 1 584.36 | 532.81 |
| 9 | w | 703.94 | 888.39 | 949.54 | 222.89 |
| 10 | w | 249.85 | 458.62 | 453.71 | 372.01 |
| 11 | woc | 1 866.81 | 1 349.81 | 2 347.98 | 1 069.86 |
| 12 | woc | 216.71 | 312.85 | 690.58 | 289.21 |
| 13 | m | 3 383.32 | 2 900.87 | 2 255.58 | 2 987.43 |
| 14 | woc | 1 260.94 | 1 429.10 | 484.11 | 1 124.06 |
| 15 | woc | 641.87 | 1 594.54 | 1 928.33 | 995.63 |
| 16 | woc | 71.68 | 141.60 | 240.27 | 168.76 |
| 17 | woc | 1 787.24 | 2 035.31 | 735.82 | 1 700.60 |
| 18 | m | 538.39 | 478.12 | 1 263.11 | 515.05 |
| 19 | w | 481.23 | 795.97 | 70.68 | 153.76 |
| 20 | m | 38.69 | 60.23 | 133.54 | 69.37 |
| 21 | m | 2 142.20 | 1 563.00 | 2 331.49 | 1 862.62 |
| 22 | w | 1 609.34 | 226.50 | 864.19 | 422.19 |
| 23 | woc | 1 567.32 | 1 330.25 | 1 774.68 | 1 184.09 |
| 24 | m | 339.13 | 271.19 | 433.92 | 167.75 |
| 25 | m | 1 481.54 | 1 374.03 | 441.68 | 270.99 |
| 26 | woc | 1.52 | 131.74 | 334.89 | 651.01 |
| 27 | m | 439.12 | 142.59 | 871.58 | 357.75 |
| 28 | woc | 680.57 | 37.08 | 1 269.95 | 277.96 |
| 29 | w | 560.52 | 257.60 | 430.12 | 311.09 |
| 30 | w | 2 007.31 | 859.78 | 1 303.32 | 435.30 |
| 31 | w | 58.76 | 97.57 | 797.45 | 257.32 |
| 32 | m | 759.56 | 238.95 | 694.96 | 154.29 |
| 34 | woc | 499.00 | 422.59 | 981.29 | 209.49 |
| 35 | m | 72.61 | 34.40 | 132.13 | 717.67 |
| 37 | woc | 48.43 | 56.91 | 424.40 | 60.86 |
|  | |  | | | |
| physical stress | | **salivary cf-nDNA [ng/ml]** | | | |
| participant | **group** | **T1** | **T2** | **T3** | **T4** |
| 38 | w | 266.40 | 157.54 | 210.91 | 295.73 |
| 39 | m | 1 223.45 | 1 394.07 | 992.15 | 2 644.14 |
| 40 | woc | 300.03 | 602.02 | 1 376.88 | 2 104.46 |
| 41 | w | 227.96 | 674.45 | 375.83 | 517.96 |
| 42 | w | 145.32 | 184.93 | 220.52 | 225.92 |
| 43 | woc | 108.70 | 84.41 | 128.78 | 135.47 |
| 44 | w | 174.66 | 488.77 | 85.98 | 209.70 |
| 45 | w | 2 104.10 | 923.25 | 1 833.29 | 1 377.68 |
| 46 | woc | 631.19 | 1 307.75 | 538.12 | 456.01 |
| 47 | w | 147.85 | 93.74 | 402.05 | 114.61 |
|  |  |  |  |  |  |
| arithmetic  mean | ng/ml | 857.95 | 792.91 | 904.35 | 812.81 |
| SD |  | 792.56 | 718.34 | 667.60 | 978.89 |

The table presents salivary cf-nDNA concentrations [ng/ml] before and after the physical stress condition. It includes participant numbers, participant groups (m = men, w = women, woc = women using oral hormonal contraception), and cf-nDNA amounts measured by qPCR at four time points: baseline (-2 min, T1), immediately after the stressor (+2 min, T2), at the expected plateau of cfDNA increase (+15 min, T3), and at the anticipated return to baseline (+45 min, T4). Given that the weight of a haploid human genome is approximately 3.23 pg, a concentration of 1 ng cf-nDNA/ml corresponds to about 310 genomic copies/ml.

**Supplementary Table 8: Salivary cf-mtDNA before and after psychosocial stress induction**

| psychosocial stress | | salivary cf-mtDNA [copies/ml] | | | |
| --- | --- | --- | --- | --- | --- |
| participant | **group** | **T1** | **T2** | **T3** | **T4** |
| 4 | m | 21 578 805 | 15 812 093 | 4 956 363 | 4 900 483 |
| 5 | w | 3 050 185 | 798 604 | 16 818 950 | 10 480 900 |
| 6 | m | 9 675 360 | 9 068 119 | 25 765 449 | 29 916 507 |
| 7 | m | 79 094 351 | 64 478 581 | 42 497 797 | 32 312 659 |
| 8 | w | 9 697 611 | 6 117 728 | 5 249 814 | 16 946 513 |
| 9 | w | 5 487 705 | 10 533 980 | 11 613 956 | 6 512 044 |
| 10 | w | 26 503 604 | 11 585 369 | 7 083 212 | 17 772 475 |
| 11 | woc | 92 587 339 | 52 694 876 | 38 199 935 | 57 822 858 |
| 12 | woc | 6 172 172 | 4 840 817 | 4 050 292 | 3 934 349 |
| 13 | m | 53 634 450 | 39 204 274 | 76 572 749 | 54 404 605 |
| 14 | woc | 29 917 856 | 25 312 275 | 8 064 162 | 19 201 671 |
| 15 | woc | 37 633 299 | 24 461 791 | 39 132 766 | 20 797 229 |
| 16 | woc | 17 835 952 | 26 020 496 | 16 613 762 | 11 446 453 |
| 17 | woc | 23 679 403 | 16 629 591 | 13 776 494 | 16 621 085 |
| 18 | m | 17 396 062 | 17 669 408 | 26 465 626 | 12 280 058 |
| 19 | w | 28 951 583 | 15 960 290 | 11 769 928 | 27 641 817 |
| 20 | m | 499 441 | 550 796 | 657 115 | 477 553 |
| 21 | m | 5 988 405 | 3 322 984 | 18 120 418 | 14 187 852 |
| 22 | w | 7 052 438 | 5 538 695 | 15 669 310 | 5 527 641 |
| 23 | woc | 92 033 961 | 40 702 615 | 7 315 383 | 37 887 544 |
| 24 | m | 2 906 367 | 1 802 450 | 2 846 109 | 1 976 032 |
| 25 | m | 2 783 051 | 6 498 971 | 3 571 983 | 3 421 343 |
| 26 | woc | 16 150 470 | 13 126 944 | 20 681 226 | 7 426 818 |
| 27 | m | 11 665 360 | 11 234 463 | 15 201 181 | 15 695 122 |
| 28 | woc | 3 244 208 | 4 111 358 | 2 765 362 | 3 564 195 |
| 29 | w | 29 814 814 | 31 421 108 | 23 384 325 | 20 670 215 |
| 30 | w | 15 991 778 | 15 012 820 | 17 940 825 | 17 098 141 |
| 31 | w | 3 500 366 | 3 724 336 | 11 097 814 | 18 476 400 |
| 32 | m | 36 233 307 | 21 658 114 | 31 697 621 | 29 210 050 |
| 34 | woc | 8 942 988 | 7 283 412 | 17 320 700 | 9 466 430 |
| 35 | m | 8 968 942 | 8 181 372 | 8 130 165 | 5 051 030 |
| 36 | woc | 5 328 798 | 14 328 335 | 6 341 645 | 4 749 800 |
|  | |  | | | |
| psychosocial stress | | **salivary cf-mtDNA [copies/ml]** | | | |
| participant | **group** | **T1** | **T2** | **T3** | **T4** |
| 37 | woc | 4 842 440 | 42 867 390 | 137 368 086 | 31 585 962 |
| 38 | w | 7 433 867 | 10 515 111 | 6 581 422 | 8 673 433 |
| 39 | m | 27 288 420 | 71 781 705 | 206 905 190 | 70 531 326 |
| 40 | woc | 18 775 278 | 37 705 939 | 241 440 546 | 102 628 106 |
| 41 | w | 13 010 153 | 33 866 006 | 34 808 732 | 31 116 509 |
| 42 | w | 14 426 168 | 11 031 079 | 7 770 300 | 2 729 913 |
| 43 | woc | 3 741 816 | 2 402 950 | 4 325 009 | 4 423 972 |
| 44 | w | 4 844 358 | 10 089 760 | 4 652 378 | 5 100 234 |
| 45 | w | 16 529 810 | 5 530 422 | 10 434 930 | 8 726 893 |
| 46 | woc | 6 996 598 | 13 786 927 | 12 873 866 | 7 546 425 |
| 47 | w | 4 293 624 | 4 308 531 | 7 617 488 | 5 043 121 |
|  |  |  |  |  |  |
| arithmetic mean | copies/ml | 19 446 115 | 17 990 067 | 28 515 125 | 18 976 367 |
| SD |  | 21 982 229 | 16 824 423 | 49 095 544 | 20 165 234 |

The table presents salivary cf-mtDNA concentrations [copies/ml] before and after the psychosocial stress condition. It includes participant numbers, participant groups (m = men, w = women, woc = women using oral hormonal contraception), and cf-mtDNA amounts measured by qPCR at four time points: baseline (-2 min, T1), immediately after the stressor (+2 min, T2), at the expected plateau of cfDNA increase (+15 min, T3), and at the anticipated return to baseline (+45 min, T4).

**Supplementary Table 9: Salivary cf-mtDNA before and after physical stress induction**

| physical stress | | salivary cf-mtDNA [copies/ml] | | | |
| --- | --- | --- | --- | --- | --- |
| participant | **group** | **T1** | **T2** | **T3** | **T4** |
| 4 | m | 9 586 751 | 7 103 060 | 7 105 661 | 5 101 368 |
| 5 | w | 3 377 125 | 6 466 021 | 1 545 799 | 2 732 145 |
| 6 | m | 23 967 073 | 19 517 701 | 22 608 982 | 30 157 497 |
| 7 | m | 26 378 077 | 17 808 810 | 16 758 325 | 53 577 778 |
| 8 | w | 13 153 519 | 9 822 836 | 26 346 229 | 9 453 114 |
| 9 | w | 21 209 513 | 27 551 200 | 17 812 495 | 12 775 245 |
| 10 | w | 5 921 214 | 9 635 235 | 4 527 895 | 6 865 818 |
| 11 | woc | 61 108 236 | 35 691 538 | 40 515 571 | 26 493 085 |
| 12 | woc | 4 642 670 | 6 132 278 | 8 265 845 | 4 775 838 |
| 13 | m | 78 214 387 | 68 814 695 | 47 731 123 | 38 573 070 |
| 14 | woc | 25 492 508 | 26 341 266 | 5 277 722 | 19 639 941 |
| 15 | woc | 26 669 136 | 63 048 150 | 69 242 584 | 18 374 772 |
| 16 | woc | 11 636 784 | 30 532 588 | 20 808 448 | 13 530 679 |
| 17 | woc | 36 017 474 | 27 478 525 | 26 175 623 | 37 328 920 |
| 18 | m | 20 530 888 | 11 028 617 | 35 417 116 | 11 664 009 |
| 19 | w | 23 508 322 | 13 021 538 | 2 850 910 | 7 433 588 |
| 20 | m | 1 350 124 | 1 442 017 | 2 390 319 | 1 940 158 |
| 21 | m | 22 173 311 | 22 780 883 | 58 373 075 | 36 534 754 |
| 22 | w | 11 624 694 | 2 398 017 | 4 230 594 | 5 154 112 |
| 23 | woc | 26 056 164 | 27 697 671 | 13 054 419 | 19 820 211 |
| 24 | m | 1 540 221 | 2 124 216 | 5 499 764 | 1 530 704 |
| 25 | m | 11 126 016 | 13 654 309 | 3 431 129 | 2 515 178 |
| 26 | woc | 20 003 752 | 8 125 435 | 8 583 775 | 16 808 640 |
| 27 | m | 8 329 753 | 6 927 080 | 11 491 564 | 12 037 632 |
| 28 | woc | 3 763 294 | 894 904 | 4 364 587 | 3 438 390 |
| 29 | w | 10 246 807 | 7 117 006 | 11 096 745 | 13 013 479 |
| 30 | w | 37 866 731 | 18 976 370 | 38 188 257 | 14 441 328 |
| 31 | w | 5 042 108 | 4 543 081 | 22 934 496 | 9 581 053 |
| 32 | m | 23 306 631 | 26 270 112 | 54 715 498 | 25 454 937 |
| 34 | woc | 21 601 988 | 21 984 430 | 27 542 576 | 8 811 403 |
| 35 | m | 2 665 277 | 1 301 924 | 3 692 008 | 4 769 388 |
| 36 | woc | 17 483 056 | 10 233 893 | 16 776 172 | 6 047 218 |
| 37 | woc | 4 729 957 | 3 712 001 | 12 044 652 | 5 998 542 |
|  |  |  |  |  |  |
| physical stress | | **salivary cf-mtDNA [copies/ml]** | | | |
| participant | **group** | **T1** | **T2** | **T3** | **T4** |
| 38 | w | 10 797 849 | 7 250 546 | 7 005 040 | 9 054 299 |
| 39 | m | 38 950 155 | 58 128 995 | 32 681 279 | 43 643 073 |
| 40 | woc | 73 529 634 | 63 590 488 | 63 601 331 | 114 180 134 |
| 41 | w | 7 856 954 | 25 287 974 | 17 875 168 | 18 186 145 |
| 42 | w | 2 442 302 | 2 480 361 | 3 613 902 | 2 537 321 |
| 43 | woc | 4 194 986 | 2 631 142 | 3 675 810 | 5 009 525 |
| 44 | w | 5 007 898 | 5 695 780 | 2 885 514 | 6 197 267 |
| 45 | w | 14 296 581 | 6 883 421 | 10 691 242 | 16 338 249 |
| 46 | woc | 8 087 797 | 11 564 513 | 2 480 174 | 6 837 228 |
| 47 | w | 6 323 317 | 4 964 837 | 8 365 062 | 5 895 484 |
|  |  |  |  |  |  |
| arithmetic mean | copies/ml | 18 414 210 | 17 410 592 | 18 704 058 | 16 610 528 |
| SD |  | 17 639 307 | 17 447 211 | 17 979 383 | 19 423 253 |

The table presents salivary cf-mtDNA concentrations [copies/ml] before and after the physical stress condition. It includes participant numbers, participant groups (m = men, w = women, woc = women using oral hormonal contraception), and cf-mtDNA amounts measured by qPCR at four time points: baseline (-2 min, T1), immediately after the stressor (+2 min, T2), at the expected plateau of cfDNA increase (+15 min, T3), and at the anticipated return to baseline (+45 min, T4).
